# Enabling biocontained plant virus transmission studies through establishment of an axenic whitefly (*Bemisia tabaci*) colony on plant tissue culture

**DOI:** 10.1038/s41598-024-73583-6

**Published:** 2024-11-15

**Authors:** Natalie S. Thompson, David Krum, Yun-Ru Chen, Mariela C. Torres, Marena A. Trauger, Dalton Strike, Zachary Weston, Jane E. Polston, Wayne R. Curtis

**Affiliations:** 1 Department of Chemical Engineering, The Pennsylvania State University, University Park, PA 16802 USA; 2 Department of Biochemistry and Molecular Biology, The Pennsylvania State University, University Park, PA 16802 USA; 3 Department of Plant Pathology, University of Florida, Gainesville, FL 32611 USA; 4 Intercollege Program in Plant Biology, The Pennsylvania State University, University Park, PA 16802 USA

**Keywords:** Geminivirus, Begomovirus, Whitefly transmission, Biocontainment, ToMoV, Whitefly host, in vitro, Plant-insect-virus interaction, Biotic, Viral infection, Biological techniques

## Abstract

Whiteflies (*Bemisia tabaci)* and the diseases they transmit are a major detriment to crop yields and a significant contributor to world hunger. The highly evolved interactions of host plant, phloem-feeding insect vector with endosymbionts and persistently transmitted virus represent a tremendous challenge for interdisciplinary study. Presented here is the establishment of a colony of axenic whiteflies on tissue-cultured plants. Efficient colony establishment was achieved by a surface sterilization of eggs laid on axenic phototrophically tissue-cultured plants. The transfer of emerging whiteflies through coupled tissue culture vessels to new axenic plants facilitates robust subculturing and produces hundreds of whitefly adults per month. Whitefly proliferation on more than two dozen plant species is shown as well as in vitro testing of whitefly preference for different plants. This novel multi-organism system provides the high-level of biocontainment required by Federal permitting to conduct virus transmission experiments. Axenic whitefly adults were able to acquire and transmit a begomovirus into tissue-cultured plants, indicating that culturable gut microorganisms are not required for virus transmission. The approach described enables a wide range of hypotheses regarding whitefly phytopathology without the expense, facilities, and contamination ambiguity associated with current approaches.

**Supplementary Information:**

The online version contains supplementary material available at 10.1038/s41598-024-73583-6.

## Introduction

Prior research suggests that up to 47% of plant disease epidemics are caused by plant viruses, with global impacts estimated at $30B in crop losses annually and 4 million in hunger-related fatalities in 2020^1^. Insects transmit more than half of these viruses, and whiteflies transmit one third of these Hemipteran-transmitted plant pathogens^2^. It is important to keep in mind that losses do not account for the crops that are not planted to avoid viral disease, either as part of conscious disease management^3^ or due to abandonment of a crop by farmers based on anticipated excessive crop loss by whitefly-transmitted viruses such as cassava^4^ and tomato^5^. The rising human population is expected to exacerbate these humanitarian implications. Experts are calling for multidisciplinary research that advances the understanding of these complex plant-insect-virus (P-I-V) interactions under permutations of biotic and abiotic stress such as climate change with associated epidemiological models and tools for surveillance, disease response, etc^4,6–10^.

Our engineering perspective on expanding P-I-V research was to identify opportunities for enabling research at a reduced cost to lower the barriers for participation. Notably, maintaining insects on plants for entomological study in growth chambers or greenhouses is expensive and prone to contamination with other arthropods and plant diseases. Previous efforts resulted in reductions in effort and cost of whitefly colony maintenance by an order of magnitude^11^. Beyond the expense of multi-organism maintenance, the mobility of insects presents a significant consideration for biocontainment. Preventing unintended pathogen release includes not only the plant virus but also the insect vector, which often utilizes multiple plant hosts and/or transmits multiple plant diseases. Notably, many of the more devastating plant viral pandemics are caused by comorbidity of multiple viruses^10^. Studies of transmissible, genetically engineered viruses further require a biocontainment facility (e.g. biosafety level 3, BSL3), which compounds the expense and inaccessibility of crucial studies of P-I-V interactions. As modern molecular biology increases access to the tools of genetic manipulation, the concerns and constraints for biocontainment inherently increase. The research described herein sought the development of an in vitro experimental system to provide a high level of biocontainment for genetically engineered insect-transmitted viruses and their host plants.

The specific subjects of this study, *Bemisia tabaci* and begomoviruses, exemplify these issues. Whiteflies have been reported to feed and reproduce on more than 500 plant species^12^ and are estimated to be responsible for 18% of plant virus transmission worldwide^8^ and $1B in annual crop losses^9^. A recent “super-abundant” increase in whitefly populations in sub-Saharan Africa is deemed responsible for up to 50% crop losses by geminivirus agents of Cassava mosaic disease (CMD^13^), and cassava brown streak disease (CBSD^14^). As a food staple for 700 M people worldwide, these losses led directly to famine^1,3^. Though more comprehensive studies are required to anticipate whiteflies potential adaptability to climate change, preliminary modeling suggests a 2 °C increase will result in an expanded range of 300–500 km^9^, exposing even more agronomic production to whitefly transmitted diseases.

Plant viruses in the genus *Begomovirus* (family: *Geminiviridae*) are transmitted by whiteflies in a persistent manner in which the encapsidated virus traverses the hemolymph to reside in the salivary glands as a basis of subsequent injection into the plant host during phloem feeding^15^. A characteristic of the *Begomovirus* genus is a highly reduced genome, where as few as six open reading frames encode the critical viral functions to facilitate replication, movement, and encapsidation. To accomplish these roles of a viral life cycle that includes translocation within both the insect and plant, the begomovirus must recruit numerous proteins from its host. For example, these viruses do not encode a DNA polymerase which must be recruited to achieve amplification of the viral genome^16^. This rolling circle replication approach has enabled a very useful tool for begomoviral study in which a partial tandem repeat of the genome (e.g., 1.5-mer) can be delivered to the plant by using *Agrobacterium* T-DNA to launch either the native or deconstructed modified forms of the virus^17^.

Of the known 1300 whitefly species, the *B. tabaci* species complex is the only known vector of begomoviruses^18,19^. Our understanding of the breadth of virus transmission may be limited by the constraints and costs of viral host range studies. As an example of this challenge, *B. tabaci* MEAM1 (Middle East-Asia Minor 1) has a particularly large geographic and host range as well as persistent and non-persistent virus transmission. An in vitro P-I-V culture system would expedite these host range screens and lower the barrier for experimental studies to understand and mitigate disease.

In the present work, we describe the plant tissue culture (PTC) methods to establish a highly compact, robust, and economical colony of axenic whiteflies (*B. tabaci*, MEAM1). The full whitefly life cycle is observed on a diverse range of tissue cultured host plants, and successful introduction and transmission of the tomato mottle virus (ToMoV) within this axenic whitefly colony is described.

## Results

### Detached egg surface sterilization

**Fig. 1:**
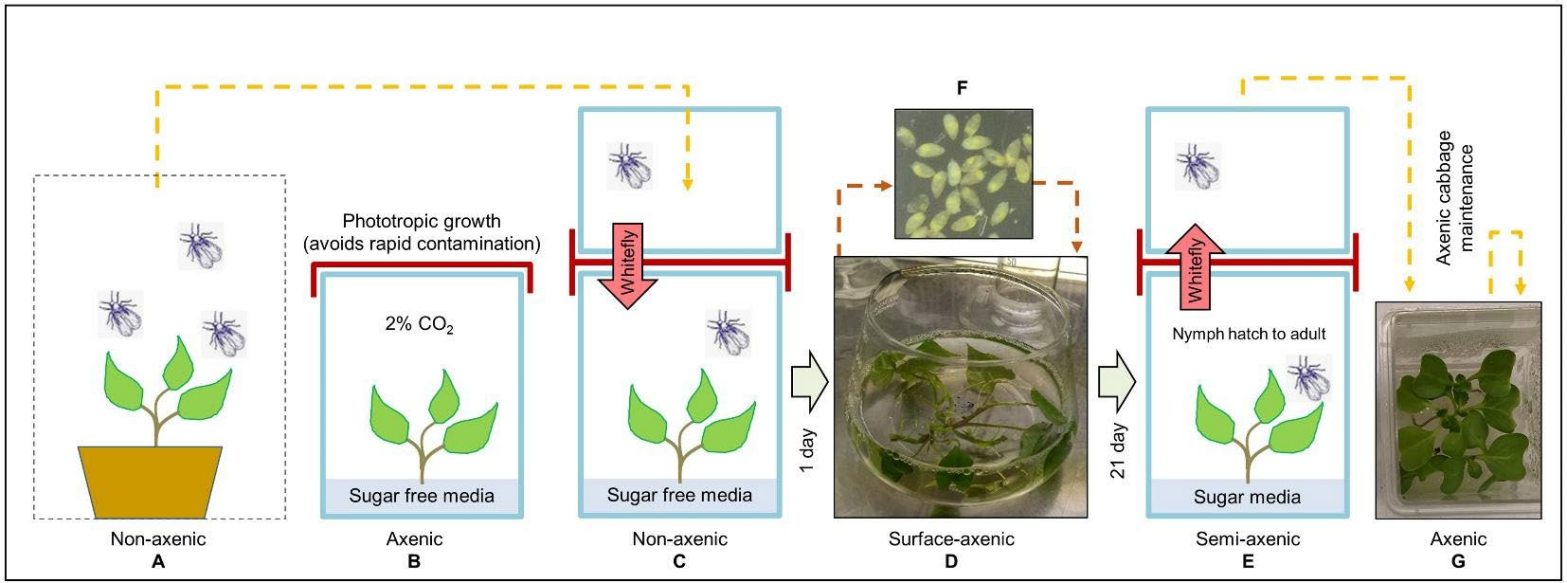
Methodology for establishment and maintenance of axenic whiteflies. **A.** Non-axenic whiteflies are collected from screen-cage colony plants and placed into GA7. **B.** Phototropically grown sweet potato grown on sugar-free media in 2% CO_2_ atmosphere. **C.** Non-axenic whiteflies lay eggs on axenic plants. **D.** Sweet potato meristems with attached non-axenic whitefly eggs are subjected to a surface sterilization procedure and placed on sugar-containing sweet potato media. **E.** Axenic whiteflies emerge after hatching, and nymphs develop in the axenic plant growth environment. **F**. Image of detached surface sterilized whitefly eggs for reference. **G.** Transfer of axenic whiteflies to axenic plants for serial monthly maintenance.

Noting that most animals require a microbiome to facilitate the breakdown and assimilation of nutrients, initial studies focused on establishing the proof-of-concept that the phloem-feeding nature of whiteflies, combined with endosymbiotic bacteria, is sufficient to satisfy the nutritional requirements in an axenic tissue culture environment. Whitefly eggs were subjected to surface sterilization after dislodging the pedicel attachment by gently brushing the underside of cabbage leaves (Fig. 1F). This approach was successful in producing emerging adults on meristem-propagated tissue cultured *Ipomoea batatas* L. (sweet potato) albeit at very low yield – likely due to a delicate balance of egg age and maturity. The axenic nature of the whiteflies was verified by absence of growth from macerated whitefly adults onto permissive R2A microbial growth agar plates^20^.

The life cycle of whiteflies at the growth temperature of 25 ^o^C is roughly 28 days^21^. Therefore, eggs laid by the primary adults that were produced from the surface-sterilized whitefly eggs require nearly a month of leaf viability to hatch and progress through the 4 nymphal stages to eclosion. Sweet potato is a vine that grows relatively quickly but was observed to excise lower leaves in tissue culture giving insufficient time for whitefly development. To overcome this limitation and allow for more efficient transfer of whiteflies to new plants, a culture device was fabricated by silicon gluing together two GA7 (i.e., clear, 3”x3”x4” polycarbonate) culture vessels with a 2-inch pass-through hole. Combining this with GA7 couplers created an L-shaped culture device where new plants could be alternately replaced (see Fig. S1.1, Supplementary Material 3, Data S1). This methodology did provide proof-of-principle with whiteflies being maintained for over a year; however, the population of whiteflies remained very low, and their appearance was sporadic. To initiate more efficient axenic whitefly establishment, an empty, inverted ‘upper’ GA7 culture vessel was installed via coupler above an upright GA7 ‘bottom’, containing a young cabbage plant. Whiteflies were collected in large numbers (100+) by exchange of the upper coupled culture vessel (see supporting video in Supplementary Material 2). This collection approach was enhanced by placing bright yellow tape on the bottom of the (clear) upper GA7 to attract emerging whitefly adults.

### Attached leaf whitefly sterilization

To expedite the initiation of large numbers of whiteflies, a GA7 of non-aseptic whiteflies was captured and coupled atop a lower GA7 containing four tissue cultured meristems of sweet potato and allowed to lay eggs (Fig. 1).

Egg laying was limited to 1–3 days by the appearance of contamination from the non-axenic whitefly honey-dew excrement. Since the whitefly eggs are securely attached to the leaf with a pedicel^22^, the sweet potato meristems could be uprooted from the agar and surface-sterilized by swirling the meristems in (cut-off) ultra-wide mouth 1 L Erlenmeyer flasks (see supporting video in Supplementary Material  1). Achieving re-sterilization of the contaminated tissue culture while maintaining viability of the eggs was constrained by systemic fungal infection that took about a week to emerge – often below the agar surface from the base of the cultured sweet potato meristem (see Fig. S1.2-A, Supplementary Material 3, Data S1). This fungus was consistent in appearance, but not identified; it is shown on potato dextrose agar (PDA (Fig. S1.2-B, Supplementary Material 3)).

A final key improvement to this procedure was to grow the sweet potato transfer host phototrophically on sugar-free media in a 2% CO_2_-supplemented growth incubator. This combination of reduced contamination during the initial egg lay, with greatly reduced rate of systemic proliferation of the fungal contaminant, allowed for the 4 weeks needed for the next generation of whitefly emergence – although issues of contamination intermittently occurred, presumably by a re-infection by feeding of emerging adults.

### Facile, abundant axenic whitefly subculture

**Fig. 2:**
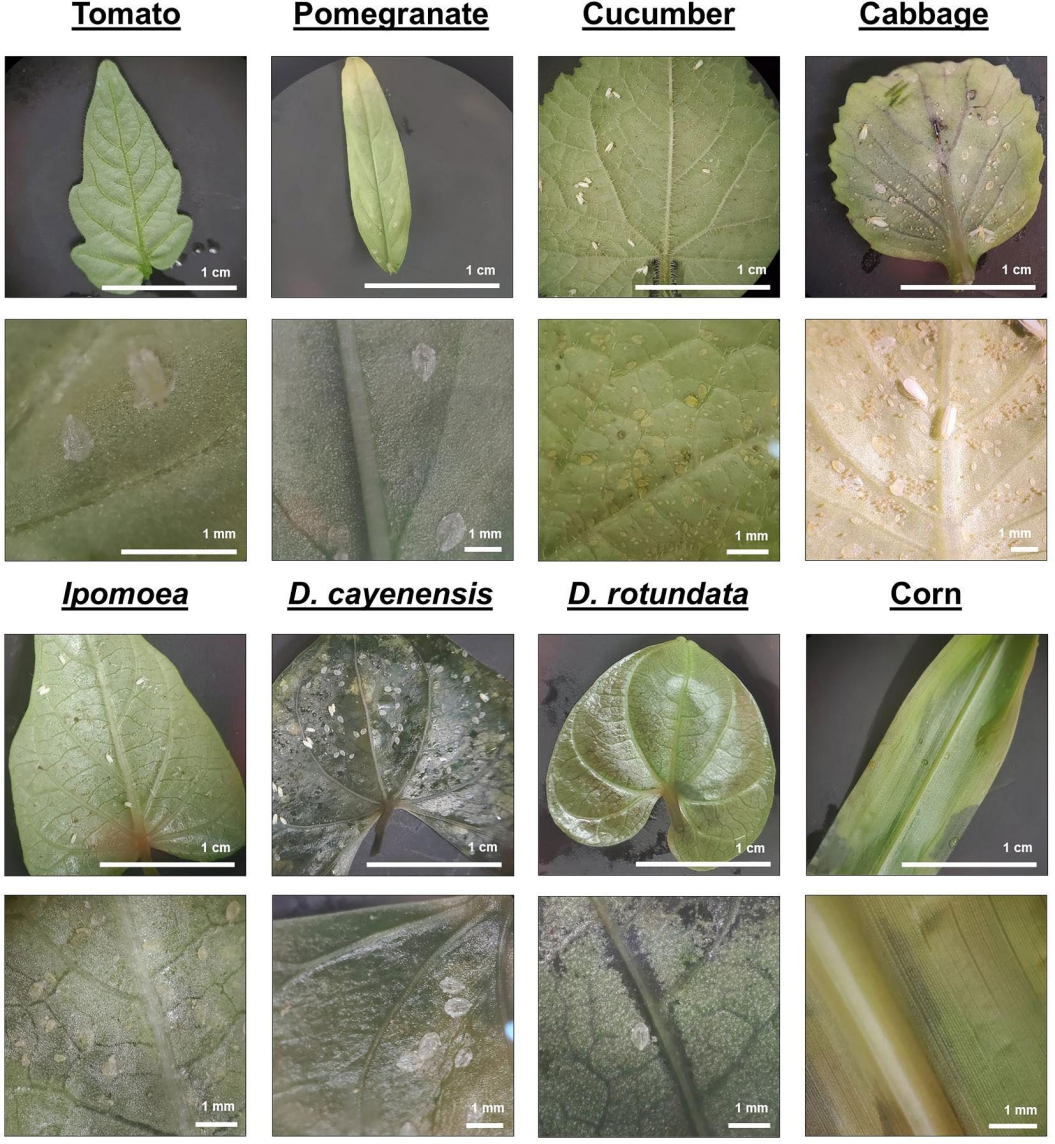
Photographs of nymph development on tissue cultured plants of *Solanum lycopersicum* (tomato), *Punica granatum* (pomegranate), *Cucumis sativus* (cucumber), *Brassica oleracea* (cabbage), *Ipomoea batatas* (sweet potato), *Dioscorea cayenensis* (yam), *Dioscorea rotundata* (yam), and *Zea mays* (corn) leaves after 30 days after initial exposure to axenic adult whiteflies. Full nymph development and adult emergence is observed on all plants except corn.

Noting that our desired tomato plant host is particularly prone to fungal infections^23^, secondary emerging adults were transferred to 2-week-old cabbage seedlings initiated from surface sterilized seeds (Fig. 1G). This resulted in a dramatic proliferation of whiteflies with dozens apparent at the top of the upper coupled GA7 (Fig. 1E) in less than a month with no fungal infections observed. Subsequent monthly subculture of whiteflies on 2-week-old cabbage provided hundreds of whiteflies to screen other plant species for their effectiveness as a maintenance host plant (MHP). Maceration of axenic whiteflies onto permissive microbial growth media confirmed the absence of culturable microorganisms, including the absence of slow-growing plant endosymbionts^24^. In screening alternative axenic plant hosts, three different assessments were made: (1) ease of PTC for given tissue culture vessel, (2) the effectiveness of the host for whitefly maintenance based on easily recoverable whitefly for transfer, and (3) observations of whitefly life cycle indicating feeding, egg lay and nymph development to adult (Fig. 2).

### Initial MHP screening

Plants such as corn, common bean, and soybean grow rapidly to very large sizes, such that the month-long culture period (necessitated by the whitefly life cycle) was difficult to contain, even in a coupled 8-inch-tall tissue culture vessel. Cowpea was more manageable, with pea providing a very convenient and compact growth habit. Okra displayed a strange behavior of pressing itself out of the agar, resulting in most of the roots out of the agar (even when germinated submerged), and sometimes inverting the plant. Tomatillo displayed a highly variable response, where its growth habit was similar to *Ipomoea* in that it rapidly grew tall with lower leaf excision. In an attempt to reduce tomatillo height, a plant in a GA7 was grown on a lighted gyratory shaker to impose thigmomorphogenesis-mediated growth height reduction^25^. Although reduction of height was minimal, there was a notable increase in leaf retention (see Fig. S1.3, Supplementary Material 3, Data S1), and more consistent production of adult whiteflies.

Numbers of seedlings in a tissue culture vessel was a significant consideration; for example, more than a single tomato seedling would result in highly crowded and epinastic growth. Recommended seed numbers are provided in Supplementary Data S3 (Supplementary Material 4) along with pictures of the plants at early stage (of whitefly inoculation) and late stage (i.e., after a month of proliferation) to give insight into their potential utility as experimental systems for whitefly-host viral transmission studies. Cucumber and radish both provided a compact plant with robust cotyledons; radish – which is also Brassicaceae – displayed overall replication numbers similar to broccoli and cabbage, both of which resulted in relatively high populations of adult whiteflies. Consistent with observations of tomato var. Lanai, another tomato variety also displayed virtually no recoverable whiteflies. An effort to obtain a ‘reduced trichome mutant’ of tomato^26^ was unfortunately not successful due to COVID disruption of breeding programs (personal communication, Dr. Dani Zamir). Other reduced tomato trichome mutants would be a high future priority for testing^27^.

Ultimately, six different prospective MHPs proceeded for further assessment of whitefly production: cabbage, *Ipomoea*, tomatillo, tomato, and two species of the viny monocot yam, *Dioscorea cayenensis* and *D. rotundata*. The two *Dioscorea* species and tomato were the intended targets for virus transmission studies; while cabbage, *Ipomoea*, and tomatillo were chosen to represent a diverse species group. Sweet potato was cultured from meristems and all other plants were grown from surface sterilized seed.

### Quantitative MHP evaluation by accumulation rates

**Fig. 3:**
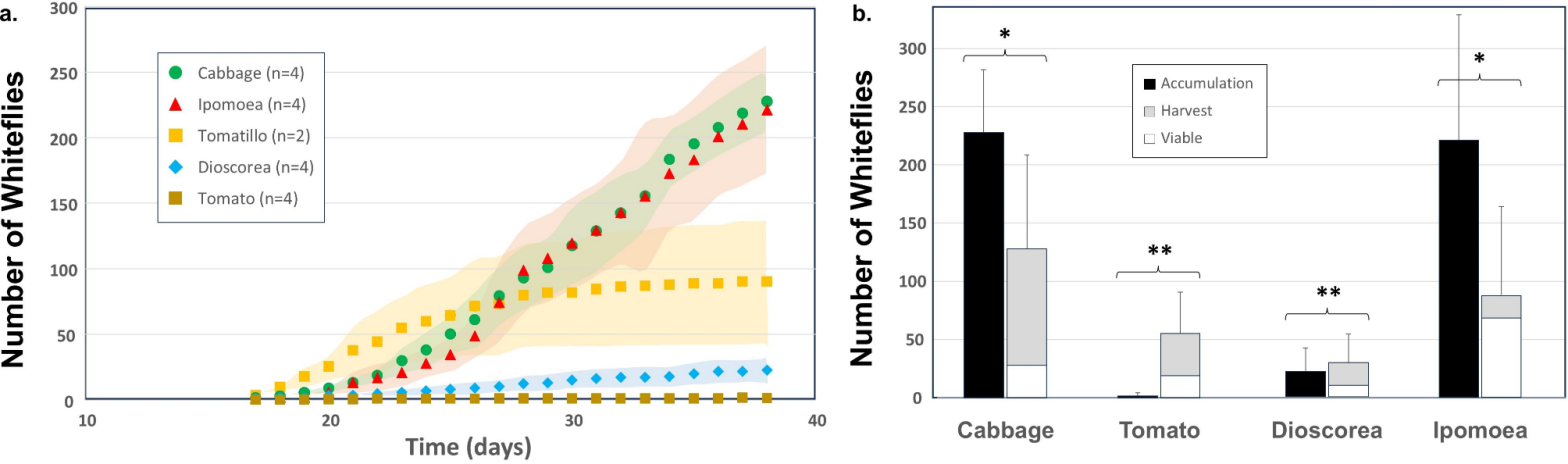
Quantitative assessment of alternative axenic whitefly host plants. (**a**) Daily accumulation based on removal from upper GA7 chamber after inoculation with 20 whiteflies on day zero. Accumulation graphs represent average of replicated experiments as provided in the figure legend. The shaded ‘confidence envelopes’ were generated based on smooth curve fit to the individual time points (α = 0.33 confidence interval corresponds to +/- standard deviation; see DataCommons file). (**b**) Total whiteflies removed during the accumulation study (black bar) and final remaining harvest counts (gray bar, n-1) for cabbage, tomato, and yam (*Dioscorea cayenensis*) and sweet potato (*Ipomoea batatas*). The number of endpoint viable whiteflies at the time of harvest (white bar overlay) is back calculated from the previously published whitefly proliferation model with further detail in Supplemental Material 3 (supplemental Fig. S1.7). Error bars are one standard deviation calculated from n-1 treatments due to one treatment being released into the cabbage viability proliferation and viability back-calculation. *Total whitefly accumulation (accumulation + harvest) is not statistically different on cabbage and sweet potato, but statistically different (**) for tomato (p = 0.0078) and yam (*Dioscorea cayenensis*, p = 0.0079).

After an inoculation of 20 ± 2 adult whiteflies and a 2-week incubation period, adult whiteflies started to emerge and fly to the upper GA7. These were captured daily to monitor accumulation rates over a six-week period (see Fig. 3A). All accumulation curves experience a ‘lag’ period of more than 3 weeks, corresponding to the egg lay and nymph development period, followed by accumulation rates that varied dramatically for different MHPs. This assessment is a useful quantification due to the need to efficiently collect and transfer whiteflies in research applications.

Tomatillo achieved a significantly higher production of whitefly when it was grown on a shaker for enhanced leaf retention due to thigmomorphogenesis. Our intended targets for virus transmission studies, *Dioscorea* and tomato, displayed essentially no whitefly capture over the entire 6-week period. As we and others have measured proliferation rates of whitefly on tomato, this observation is clearly an artifact of the PTC environment and/or the accumulation method. The compact trichome density in tissue cultured tomato is a possible explanation of poor proliferation as well as simply avoidance of leaving the underside of the tomato leaves (see Harvest results below). As is clear from Fig. 2, whiteflies clearly proliferated on these hosts, with prolific eggs, nymphs and exoskeletons of 4th instars nymphs indicating emerged adults (see Fig. S1.4, Supplementary Material 3, Data S1 for additional MHP screen photos).

### Post-accumulation harvest

**Fig. 4:**
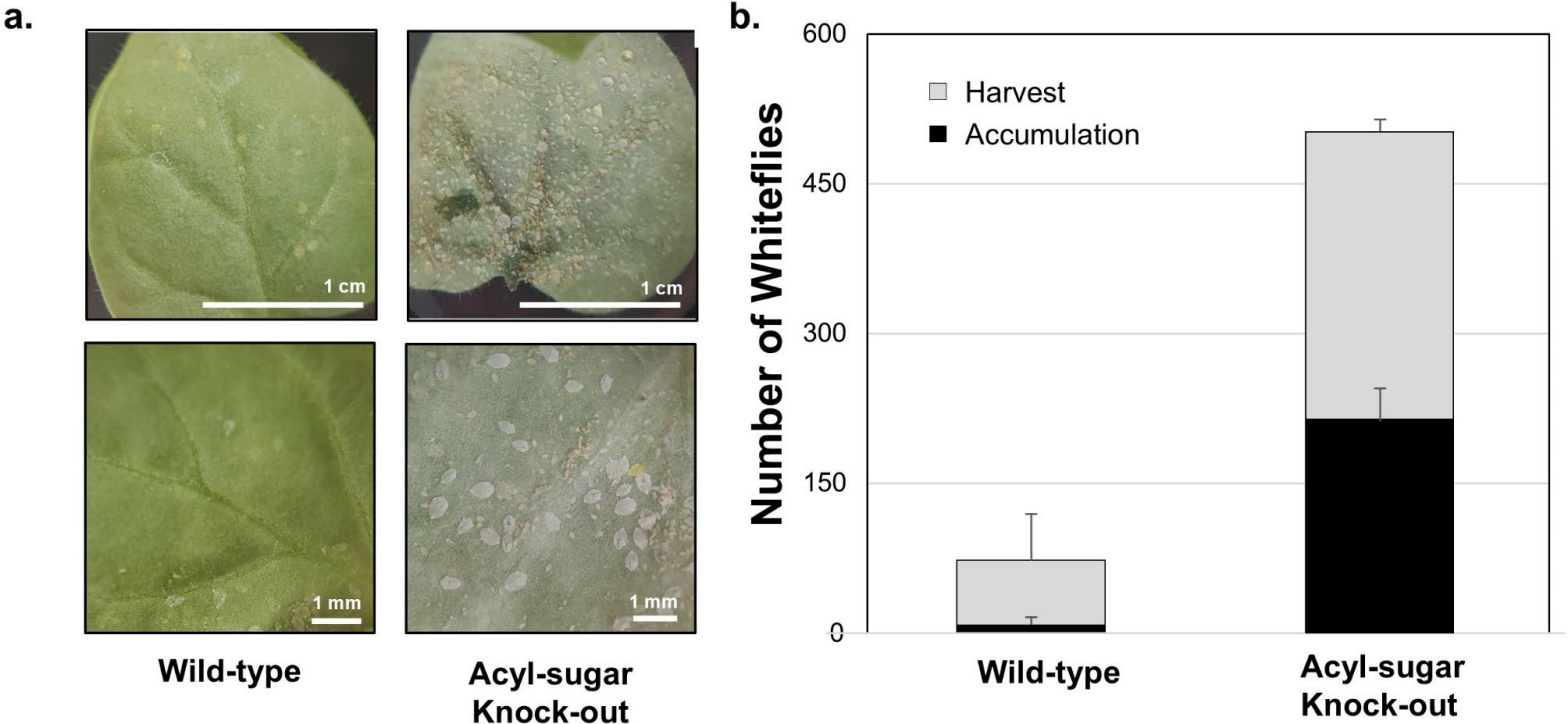
Demonstration of improved whitefly compatibility with model plant *Nicotiana benthamiana* for an acyl-sugar knockout (ASKO). (**a**) Photographic comparison of whitefly proliferation on wild-type and ASKO after 6 weeks of proliferation from 20 initial whiteflies. (**b**) Quantitative comparison of total proliferation measured as the sum of accumulation removed daily from a coupled GA7 and the remainder at harvest endpoint counted by hand. Error bars represent a standard deviation where the improved whitefly proliferation for ASKO was highly statistically significant: whitefly accumulation (*p* = 0.0005), harvest (*p* = 0.0052), and the sum total (*p* = 0.00011).

Noting that accumulation via the upper coupled GA7 is a functional measure of the utility for experimental colony maintenance—rather than a direct measure of proliferation—a destructive harvest was undertaken for cabbage, tomato, *D. cayenensis*, and sweet potato (*Ipomoea*) (Fig. 3B), and acyl-sugar knockout (ASKO) *Nicotiana benthamiana (N.b.)* (Fig. 4).

At the end of the 6-week accumulation period, all but one of the four experimental replicates were harvested for examination of total whitefly proliferation. After placement of the GA7 culture vessel in a cold room (4 °C) to immobilize all remaining whiteflies, the contents (plants, black-sand coated agar, and walls) were carefully examined to count the total final adult whitefly harvest. Both cabbage and sweet potato revealed an additional ~ 100 whiteflies present at the end of the 6-week cultivation period for a total of about 300 adult whiteflies and corresponding developing nymphs. Figure 3B summarizes the overall total of proliferated whiteflies over the 6-week period including accumulated, harvested, and an estimate of viable whiteflies at the time of harvest (see below). Cabbage and *Ipomoea* (SW) provided the most whiteflies, with cabbage providing more consistent proliferation between replicates. It is not surprising that *Ipomoea* can act as a host since the common name of our vector is ‘sweet potato whitefly’. These overall proliferation totals suggest the maximum capacity of this culture system when whitefly proliferation is non-MHP-limited. This final harvest approach provided additional insight into the limitation presented by tomato and *D. cayenensis*, where both display considerable proliferation beyond the inoculum that were not recoverable via accumulation capture.

For the wild-type *N. benthamiana* (Fig. 4B), whitefly numbers more than doubled from the initial inoculum, while the vast majority of the whiteflies remained associated with the plant and the culture vessel. Notably, many whiteflies were stuck onto the leaf upper surface that was not apparent for the *N.b.* acyl-sugar knockout (ASKO). Whiteflies displayed a greatly improved proliferation on the acyl-sugar knockout of *N. benthamiana* as compared to wild-type (Fig. 4). This comparison is particularly interesting because of the alternative hypothesized insecticidal roles of acyl-sugars as either mechanical (stickiness of trichomes) or chemical (metabolic disruption)^28^. An end-point harvest was performed after the 6-week accumulation study as above (Fig. 4B). Consistent with a several-fold higher accumulation and final harvest, *N. benthamiana* ASKO displayed far fewer whiteflies ‘stuck’ to the top surface of the leaves compared to wild type *N. benthamiana*. A method of entrapment of the initially inoculated flies would contribute to the greatly reduced whitefly on the wild-type plants. These observations support the conclusion of the physical role of acyl-sugar stickiness in capturing whiteflies.

### Viability assay

For further assessment of MHPs, the viability of the whiteflies at the end of the accumulation time course was assessed using a quantitative fecundity bioassay on cabbage as described elsewhere^11^. In brief, the GA7 tissue culture vessel was introduced into a screen-cage to release the whiteflies onto cabbage to allow proliferation on this favorable host plant. After an additional six weeks of proliferation, the whitefly amplification was assessed using image analysis, and then the initial viable whiteflies needed to give rise to the bio-assay quantified whitefly count was back-calculated using our previously parameterized predator-prey model for this experimental condition. This approach provided for a quantitative estimate of the corresponding number of flies that were healthy and viable on the different MHPs at the end of the accumulation period (Fig. 3B). These results clearly confirm that ease of recovery of proliferating whitefly for experimentation varies significantly for different plant hosts.

### MHP whitefly preference comparisons

**Fig. 5:**
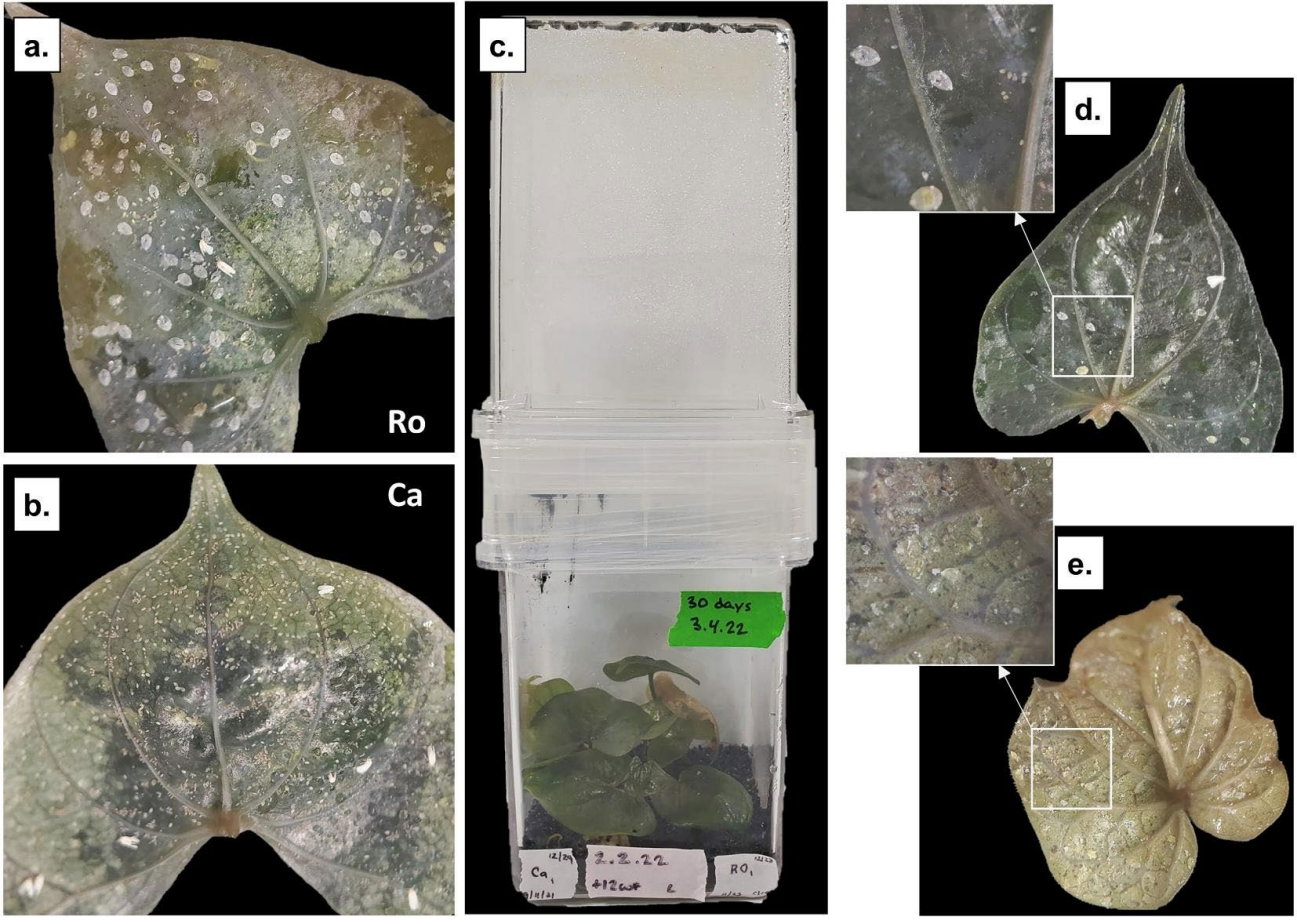
Preference studies with the axenic colony. Two species of the monocot Asian yam – *D. rotundata (* **a**) and *D. cayenensis* (**b**) were propagated in the same GA7 (**c**) and after a 30-day proliferation period, images of nymph development were taken. Similar preference studies were undertaken comparing *D. rotundata* (**d**) to dicot sweet potato *Ipomoea* (**e**).

With a fully axenic colony, direct comparisons of insects’ preferred MHP are straightforward to implement – although subject to in vitro interpretation. *D. cayenensis* and *rotundata* were subcultured into the same GA7 (see Methods); whiteflies were then added and after a 30-day proliferation period, qualitative comparisons of nymphs and emerged exoskeletons that had developed on each plant species were possible (Fig. 5A-B). When provided both hosts simultaneously in the same vessel, whiteflies displayed a distinct preference for the *D. rotundata* over *D. cayenensis*. A further test comparing *D. cayenensis* to *Ipomoea* within the same GA7 were consistent with accumulation study results of the preference of *Ipomoea* (Fig. 5D-E).

### Axenic virus transmission

**Fig. 6:**
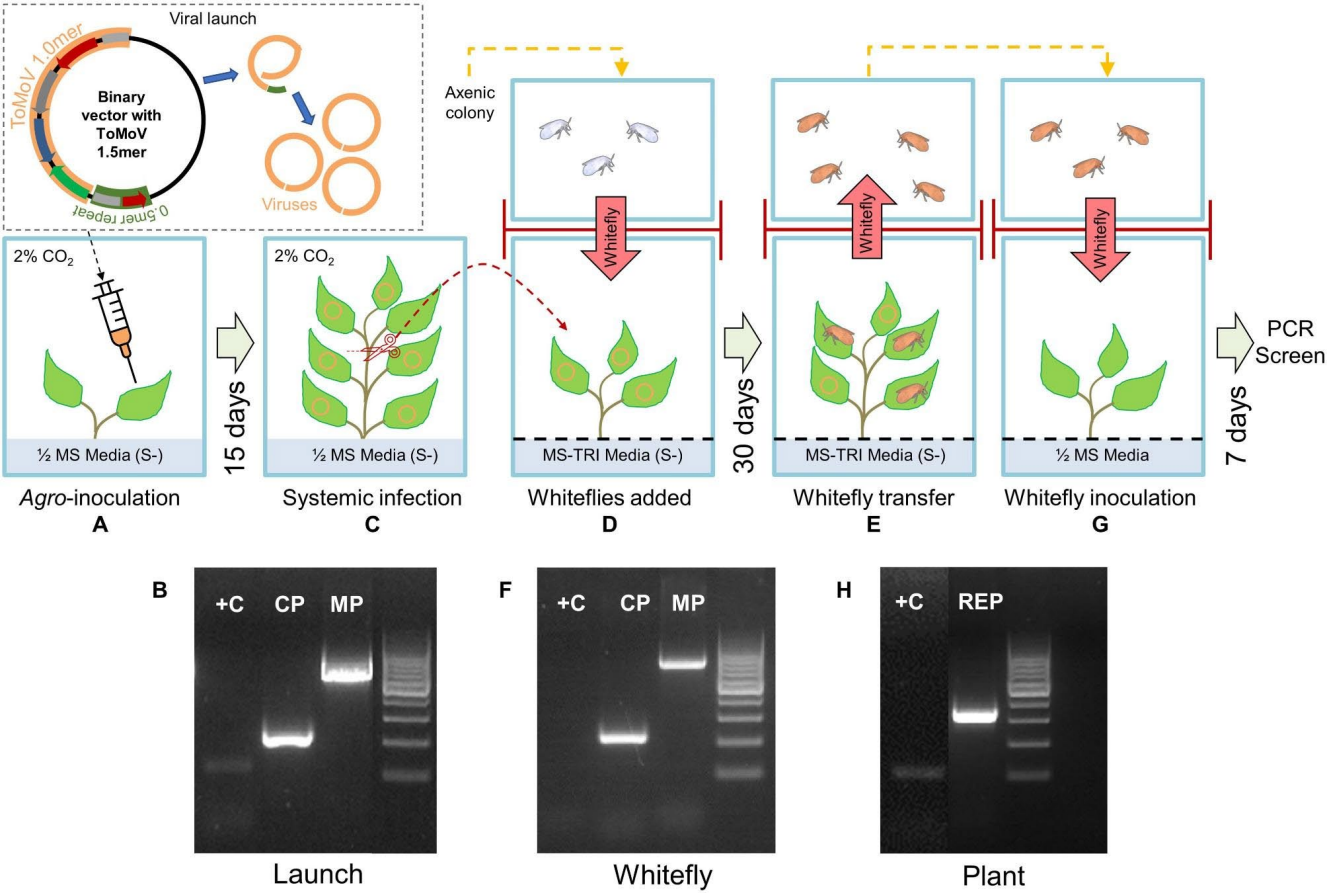
Methodology for in vitro virus transfer with axenic whiteflies. (**A**) Axenic ASKO *Nicotiana benthamiana (N.b.)*, grown on sugar-free media supplemented with 2% CO_2_ to encourage phototrophic growth, syringe infiltrated with *Agrobacterium* containing the ToMoV 1.5mer infectious clone to launch a viral infection (depicted in upper left panel). (**B**) 15 days after syringe infiltration, systemic infection was confirmed via PCR (along side a GeneRuler 100-bp ladder): CP = coat protein (227-bp), MP = BR1 movement protein (719-bp), and + C weakly positive control for transgenic plant NptII (112-bp). (**C**) upper new systemic plant growth was aseptically excised, and (**D**) placed onto Root Induction Media (MS-TRI) with black sand covering the agar to protect whiteflies; whiteflies were then added from the axenic colony. (**E**) 30 days after whitefly addition, newly emerged adults containing the virus were captured. (**F**) Whitefly were screened by PCR for the presence of the virus (CP and MP); -C negative control for absence of transgenic plant NptII (**G**) Viruliferous whitefly transferred to a newly grown *N.b.*(ASKO) in aseptic tissue culture. (**H**) 7 days later, leaf samples were PCR screened for the presence of the virus. Orange circles on plant leaf represent virus; orange whiteflies represent viruliferous. Note that gels B and H were cropped to provide ease of interpretation and align adjacent molecular markers. Original uncropped gels are provided as Supplementary Material 3, Data S1.8.

A major motivation for establishing an axenic whitefly culture was the goal for a biocontained experimental system for the study of plant viruses – in this case, whitefly mediated virus transmission of a begomovirus. Axenic whiteflies fed on virus-infected *N.b.*(ASKO) plants for a 72-hour minimum acquisition access period (AAP). The whiteflies were confirmed to contain both the A- and B-component of the virus based on amplification of coat and movement protein genes respectively (Fig. 6). The viruliferous axenic whiteflies were then moved to fresh axenic *N.b*.(ASKO) After a one-week inoculation access period (IAP) on the fresh plants. Mottling was observed on the target plants, and although this is indicative of virus transfer, the tissue culture environment for *N. benthamiana* seedlings can produce a somewhat mottled appearance which limits this visual confirmation. The presence of the ToMoV virus in the target plant was further substantiated by PCR amplification of the REP gene, which was then gel extracted and Sanger sequenced (Fig. 6H).

## Discussion

A bench-scale protocol for establishing axenic whitefly (*Bemisia tabaci*) was achieved, which involved a combination of surface sterilization and phototrophic plant tissue culture techniques, using readily available off-the-shelf components. The method’s robustness was tested with 27 plant species, including monocots and dicots, converging on cabbage as an optimal maintenance host plant (MHP) based upon whitefly proliferation rates; in turn, whiteflies were easily maintained for over two years via serial subculture with minimal expense and monthly maintenance requirements.

The observed successful whitefly proliferation across a broad range of axenic maintenance host plants (MHPs) suggests that for tissue cultured plants there are not nutritional limitations for using *B. tabaci* MEAM1 in studies of other plant species. A key characteristic of an effective PTC is that the leaves on which eggs are laid must stay healthy for at least one month. Seedling-based PTC seems to be particularly effective for plants with highly developed cotyledons during seedling establishment (e.g., cabbage, pea). Although we experienced issues with larger seedlings (in GA7s), tissue culture vessels are available to accommodate larger plants. It is possible to manipulate plants in tissue culture with plant hormones (e.g., auxins, cytokinins) to improve specific plant host effectiveness. For example, recent methods of stunting corn (*Zea mays*) with gibberellic acid inhibitor ancymidol^29^ to facilitate transformation would allow more careful study of this critically important global crop species which has recently been reported as an emerging whitefly host^30^.

Implementation of this method facilitated preliminary axenic studies of insects’ feeding preference between different plant species. Whiteflies in this system preferred *D. cayenensis* over *D. rotundata*. Meanwhile, the fortuitous recent development of the *N. benthamiana* acyl-sugar knoclout (ASKO) provides an exceptional platform on which to conduct future studies of begomoviruses and their insect vectors^28^. *N. benthamiana* has been demonstrated to serve as a very broad host for plant viruses and is also the model plant for rapid gene expression using *Agrobacterium*, particle bombardment, and nanoparticle delivery^31–33^. The transgenic CRISPR-Cas9 / TRV system provides for rapid (homozygous) knockouts^34^, which could facilitate examination of the roles of specific plant genes involved in the virus recruitment of plant functions to achieve its pathogenicity. Our preliminary observations of whitefly proliferation in *N. benthamiana* ASKO suggest that the primary role of acyl-sugars in protection against whitefly is not related to insecticidal action or fecundity but more likely to the ‘stickiness’ that visibly captures adult flies which then die from desiccation.

Transmission studies of plant viruses, and particularly genetically engineered or more virulent biotype viruses with insects, represent an important biocontainment challenge. Restrictions such as BSL3 containment for viral transmission are cost-prohibitive, particularly for most agricultural funding. The extra level of biocontainment provided by axenic viral transmission enabled acceptable protocols by USDA-APHIS for BSL2 transmission of recombinant viruses. This creates opportunities for plant viral study that would otherwise be considered insufficiently contained and/or too costly for academic researchers. Equally important is the simplicity and cost efficiency of this axenic whitefly culture system which can be implemented in a few square feet of non-specialized laboratory space for those with general plant tissue culture experience. The materials and supplies to maintain the axenic colony on cabbage seed are insignificant for any laboratory capable of tissue culture – requiring only a dozen tissue culture vessels including the active colony and tissue culture host plant. Preparation of media, MHP seed sterilization, and whitefly transfers require about one person-day per month with minimal monitoring and maintenance. In particular, the time-consuming daily maintenance of plants is eliminated.

Although the use of *Agrobacterium* to launch a virus into tissue cultured plants provided a proof-of-principle for the transmission of ToMoV and achieved a highly biocontained procedure for virus introduction into the axenic whitefly colony, it has limitations for extension to other viruses. We therefore sought to develop alternative approaches to introducing filter sterilized intact virus to the whitefly. Filter sterilization of the virus extract was accomplished using a 0.22 μm PTFE syringe filter. Many viruses can be isolated in an encapsidated form which facilitates feeding to the whitefly through the ‘sachet method’ of droplets sandwiched between layers of parafilm stretched thin^35^. We demonstrated that the wax film could be sterilized with a 10-Gy dosage gamma irradiation treatment from a Cobalt-60 dry cell gamma irradiator. Since the gamma irradiation treatment imparted brittleness to the parafilm it was necessary to stretch the film prior to irradiation. This aseptic sachet method was used to feed a simple plant extract from a ToMoV-infected tobacco plant into the axenic whitefly colony to transmit the virus to plant tissue cultured ASKO *N. benthamiana* as confirmed by PCR. Additionally, artificial feeding of whiteflies through autoclavable membranes has been reported in a brief description^36^. Since this approach would be more broadly accessible, we identified a readily available autoclavable PFTE asymmetric membrane and confirmed whitefly feeding based on uptake of a fluorescent feeding solution containing 150 g/L sucrose and 10 µM Texas Red dye. The feeding device details, including implementation into a GA7 plant tissue culture lid, are described in Supplementary Data S4 (Supplementary Material 5).

The presence of whiteflies feeding on tomatoes was previously shown to cause systemic proteome expression changes^37^. Infection by the tomato mottle virus (ToMoV) was shown to suppress characteristics of the defense response, suggesting that ToMoV may benefit its whitefly vector by masking its feeding. Our observations of systemic host plant fungal infection due to non-axenic whitefly feeding, however, provide an alternative interpretation where this systemic response could be mediated indirectly by vascular system contamination. The axenic cultures developed here could provide clarity of this observation. The nature of phloem-feeding insects results in non-viral microbial contamination that can also be resolved using an axenic virus transmission system. The methods described for whitefly egg handling also present the opportunity for clearing an insect colony of non-vertically transmitted viruses.

The membrane feeding methodology described herein enables improved whitefly RNAi silencing such as typical innate immunity studies^38^. These innate immunity assays of whiteflies with non-axenic sachet feeding typically involve off-target effects from contamination^39^, resulting in a competition between targeted physiological response and death from septic shock. In this axenic system, the off-target effects of bacterial contamination can be eliminated. Execution of such RNAi studies using an axenic feeding system could improve stability of delivered interfering RNA and reduce the detrimental impact of artificial feeding. Combined with virus-like particles, the delivery and vitellogenin targeting of CRISPR might be accomplished through axenic membrane feeding. As a simplistic but no less important method, an axenic culture may provide a means of developing an artificial diet with fewer off-target effects.

The absence of culturable gut microbiome which enabled the establishment of this tissue culture system, provides an additional level of dissection of this complex P-I-V interaction. PCR was used to compare the presence of the typical whitefly endosymbionts in the axenic and non-axenic whitefly colony. This included the primary endosymbiont *Portiera aleyrodidarum*, along with *Hamiltonella*,* Arsenophonus*,* Cardinium*,* Wolbachia*,* Rickettsia*,* and Fritschea*. The PCR primers used for these endosymbiont amplifications are listed in Supplementary Material 7, Data S7^40–46^. *Rickettsia* and *Hamiltonella* were the only secondary endosymbionts present in the axenic colony. Interestingly, a PCR screen for the primary endosymbiont presented the unexpected drop below the detection threshold for *Portiera* in the axenic culture that recovered when the axenic whiteflies were released back to non-axenic culture on cabbage plants (see Fig. S1.5, Supplementary Material 3). This result suggests substantial alteration in endosymbiont populations and may also allow deciphering their roles in whitefly biology. For example, it has been reported that GroEL provided by the endosymbiont, may be required for persistent virus accumulation in the whitefly salivary glands^47^.

Inexpensive deep sequencing is revealing large numbers of asymptomatic ‘non-culturable’ viruses which have potential to be harnessed for beneficial plant improvement or represent a reserve of future viral pathogens^6^. Noting that this research was initially funded by the DARPA Insect Allies program, use of insects to deliver beneficial crop protection included a requirement of achieving control over the whitefly vector. A tissue-cultured plant host provides for the potential development of biocontainment conditional lethals such as the tetracycline repressor (Tet-off), which when linked to lethal genes has been used for other insect conditional sex-specific lethality^48,49^. Given the phloem-feeding nature of whiteflies, combined with their extended development as phloem-feeding nymphs, the repression of an insecticidal trait would require the presence of the repressor from the MHP throughout the insect life cycle; the potent anhydrotetracycline repressor might provide such repression^50^ at levels far below general biological toxicity. Transgenic methods such as lethal gene silencing and toxin-antitoxin may provide a means of inducing rapid death to a whitefly host that escapes the tissue culture environment.

In summary, the methodologies and techniques described herein enable the establishment of a fully axenic whitefly colony. Refinement of this method facilitated an axenic Plant-Insect-Virus virus transmission protocol, through successful demonstration of serial transfer of (bipartite) ToMoV within the axenic whitefly colony. The communal axenic nature of this system provides an experimental platform that minimizes the influence of other microbes in studies of plant-virus-vector interactions.

## Methods

### Whitefly colony

*B. tabaci* MEAM1, from a colony started in the early 1990s with adults collected from field tomato plants at Univ. of Florida, Gulf Coast Research and Education Center, Bradenton, FL. Whiteflies were maintained in growth chambers on cabbage during winter months and on cotton plants during the summer months^51^.

### Plant sourcing & seed sterilization

Screening of additional alternative hosts was undertaken by introducing roughly a dozen whiteflies (reared on cabbage) to tissue-cultured plants established by surface sterilization of seeds. Seeds used for axenic screening of whitefly proliferation included varieties from Livingston Seed (https://livingstonseed.com/): radish (*Raphanus sativus*, crimson giant), pepper (*Capsicum annuum*, jalapeno, California wonder), okra (*Abelmoschus esculentus*, Clemson spineless), pea (*Pisum sativum*, Lincoln), cowpea (*Vigna unguiculata L.*, California blackeye), cucumber (*Cucumis sativus*, straight eight), cabbage (*Brassica oleracea*, red acre heirloom), ornamental corn (*Zea mays*, Indian corn), broccoli (*Brassica oleracea*, green sprouting Calabrese), zucchini (*Cucurbita pepo*, golden), and tomatillo (*Physalis philadelphica*). The cabbage varieties of ‘Earliana’ and ‘All Seasons’ were obtained from W. Atlee Burpee and Co., Warminster, PA. Other plant species seeds were sourced from local market dumpsters such as: lemon (*Citrus limon*), orange (*Citrus sinensis*), and pomegranate (*Punica granatum*). Optimal counts of seeds depending on plant species per GA7 are denoted in Supplementary Material 4, Data S3. Seeds were surface sterilized for a range of conditions as necessary to prevent contamination when germinated on ½ strength MS media^52^: 10–15% v/v commercial bleach (6.0 wt% Na-hypochlorite) with 2–3 drops of Tween-20 (Sigma, CAS: 9005-64-5) for 10–15 min on a gyratory shaker (New Brunswick Scientific Co., Gyrotory^®^ Shaker-Model G2, 40 RPM), followed by 3x rinses with sterile distilled water. Virus-indexed, tissue-cultured sweet potato (*Ipomoea batatas*) var. Beauregard and meristem maintenance procedure^53^ were kindly provided by Christopher Clark, Dept. Plant Pathology & Crop Physiology, Louisiana State University and serially propagated on SW media (Supplementary Material 6, Data S6, with and without sucrose as noted). *Dioscorea cayenensis* (Asian yam) was provided by Morufat Balogun, University of Ibadan, Nigeria. Plantlets of *D. rotundata* (IITA accession no. Tdr2436) were provided by Leena Tripathi, IITA-Nairobi, Kenya. Tomato (*Solanum lycopersicum*) plants var. Florida lanai were generously provided by Dr. Jane Polston, University of Florida.

### Routine plant tissue culture

PTC and an axenic whitefly colony were maintained in a standard laboratory room designed to provide roughly 25 ^o^C with large numbers of plant culture growth racks with a 16:8 diurnal light cycle, PAR levels of roughly 110 µmoles m^−2^ s^−1^, and the GA7’s roughly 6 inches from light. *Dioscorea* Yam Basic Medium (YBM) is described previously^24^. Subculturing was ideally executed monthly but could be delayed to up to 6 months if GA7 tissue culture vessels were sealed with strips of plastic wrap to prevent water loss.

### Quantitative kinetic studies

The qualitative studies identified the methods for handling of different plant species where cabbage and sweet potato performed very well, and tomato and Asian yam (Dioscorea) performed poorly. Tomatillo was included in these studies since it can serve as a viral host that is related to tomato. While working in a laminar flow clean bench, whiteflies were captured by placing a GA7 coupler on top of the cabbage maintenance tissue culture and was removed when 20 ± 2 whiteflies had moved into the upper polycarbonate vessel. The addition of a piece of yellow tape was useful to promote whitefly movement. After confirming the exact number captured in this GA7, the coupler was transferred to the experimental plant treatment – where all flies would move to the plant within a day. These coupled GA7 vessels were monitored for the appearance of whiteflies in the upper GA7 which would occur after the emergence of the next generation of whiteflies after a 2-week period. The upper GA7 would then be checked daily and replaced when whiteflies were removed. This removal of mobile whiteflies was repeated for 3-weeks where the accumulation was tabulated as the sum of daily whitefly removal.

### Harvest method

After placement of the GA7 culture vessel in a cold room (4 °C) to immobilize all remaining whiteflies, the contents (plants, black-sand coated agar, and walls) were carefully examined to manually count the total final adult whitefly harvest– where total harvest count reported included subtracting the initial whiteflies added.

### Preference studies

Qualitative preference studies included growth in modified GA7 culture vessels to accommodate two different plant species in the same vessel. Comparisons included two different yam varieties (*Dioscorea cayanenes*, and *D. rotundata*) and yam versus sweet potato variety noted above. Notably sweet potato plants performed very poorly for attempted growth on *Dioscorea* YBM media which required two media in the same growth container. Therefore, preference studies were initially conducted with a constructed GA7 that contained a thin wall of polycarbonate dividing the bottom into two compartments. The wall was affixed to the container with silicone caulk which was autoclave compatible. In testing however, based on aberrant plant growth, the caulk was sufficiently permeable to allow the hormones to travel across the barrier. The next iteration successfully used a plastic cap of a 25 mm culture tube as a “container” to hold media/hormones for one plant (e.g. SW media for sweet potato) with alternative media in the GA7 outside the cap (e.g. YBM media for yam).

### Viability derived from kinetic model

Since the total fly harvest method does not distinguish the healthy whitefly population from the dead/dying whiteflies at the harvested endpoint, we used a kinetic bioassay model to back-calculate the viable whiteflies^11^. This deterministic kinetic model was based on non-axenic whitefly colony proliferation characterized by image analysis (ImageJ, 64-bit Java 1.8.0_172) of the cabbage plant surface area as well as automated counting of whiteflies after 6 weeks of proliferation. Briefly, a GA7 from the end of the 6-week accumulation time course was opened in a screen cage with a healthy cabbage plant (see Fig. S1.6, Supplementary Material 3). Upon desiccation of the tissue-cultured plants, the viable whiteflies transferred to the cabbage host. If the number of transferred whiteflies was high, another cabbage plant was added to the cage after 4 weeks to maintain a reasonable stress on the plants as required for the whitefly proliferation predator-prey type model. In this manner, the number of total whiteflies harvested after 6-weeks could then be used to back-calculate the number of viable whiteflies from the GA7 being tested.

### Egg sterilization

The whitefly eggs were surface sterilized according to existing protocols of a dilute bleach contact, followed by sodium thiosulfate neutralization and rinsing^54^.

### Axenic whitefly maintenance

Establishment of the axenic whitefly colony ‘evolved’ over a period of several years as described in Results. Routine maintenance of the axenic whitefly colony was ultimately achieved by monthly subculture using six surface sterilized seeds of cabbage (Burpee, ‘All Seasons’) in a GA7 polycarbonate 3 × 3 × 4-inch culture vessel (Magenta; magentallc.com) containing ~ 50 mL of ½ MS media. Seeds were sown ~ 2 weeks prior to the monthly whitefly transfer, where autoclaved black aquarium sand (Imagitarium, Aquatic Substrate) was added to reduce whitefly death by ‘drowning’ on the agar; sand was added about 2 weeks after germination to verify the absence of contaminant growth. Whitefly transfer is accomplished using a GA7 coupler (Originally Magenta, LLC; now available as SPL Life Sciences, Cat. No. 310071) that allows bringing two vessels together (see video in Supplementary Material 2). After ~ 3 weeks of proliferation, dozens of whiteflies will accumulate within the upper chamber of the stacked GA7s. By swapping out a new sterile GA7 and flipping it over (with addition of a piece of yellow tape for attraction), the whiteflies transfer into the new GA7 that has minimal condensate which is then placed on the next tissue cultured plant.

### Whitefly membrane feeding apparatus

We identified a source of autoclave sterilizable membrane material (TISCH, https://scientificfilters.com/, cat# RS40113, PFTE, 1.0-micron pore size). A sterilizable tissue culture lid with feeding membrane was fabricated by placing disks onto the end of a ¾”ID /1”OD acrylic tube (US Plastics, www.usplastic.com, cat #43109) and secured with silicone glue over a hole drilled in the polypropylene GA7 lid (Fig. S4.1, Supplementary Material S5, Data S4). Feeding was verified by placing 10 µM Texas Red dye (Fisher Scientific, sulforhodamine 101 acid chloride, cat# 506355805|AAJ60581MA) in a 150 g/L sucrose solution and feeding in a dark room illuminated with red light to avoid dye photobleaching.

### Virus agro-infection preparation

*Agrobacterium tumefaciens* (Cys32)^55^ containing infective T-DNA clones of tomato mottle virus (ToMoV) for both A (pMon512//ToMoV{A,1.5mer}) and B (pLSU-1//ToMoV{B,1.5mer}) components of the bipartite virus were grown at 28 °C for 36 h until an OD_600_ of 0.8 was reached. Updated sequences for these viral genome sequences were generated by Next-Generation sequencing (plasmidsauris.com) and are provided as GenBank sequences (accessions A: OR392453 and B: OR392454). Both the A and B cultures were then pelleted and resuspended in infiltration buffer (10 mM MgCl_2_, 10 mM MES pH 5.6, 100 ${\mu}{\text{M}}$ acetosyringone) to an OD_600_ of 0.6. The two cultures were combined at equal volume ratios and incubated for 2 h at 28 °C. Following this, a surfactant (Silwet L-77, Lehle seeds, Cat. No. VIS-01) was added to a concentration of 0.02%.

### Virus transmission studies

The *N. benthamiana* ASKO seeds were kindly provided by Dr. Georg Jander from Boyce Thompson Institute, Cornell University. These *N.b*. (ASKO) seeds were kanamycin resistant which served as a convenient PCR control. *N.b.* (ASKO) seeds were surface-sterilized and grown on ½ MS media without sucrose in a 2% CO_2_-supplemented atmosphere to promote phototrophic growth until the first primary leaves developed (~ 3 weeks). The A{1.5mer} and B{1.5mer} *Agrobacterium* infective clone cultures were syringe (BD 3 mL Syringe, Luer-Lok Tip, Ref: 309657) infiltrated into the lowest fully developed leaves. At 11 days post-infection, top systemic leaves were assessed for presence of the virus via an Edwards DNA extraction followed by PCR screen (details below). Following confirmation of viral infection, explants were cut from above the infiltration site and placed in root inducing media (MS-TRI, Supplementary Material 6, Data S6) with two nodes per explant. After a period of recovery and growth, 10 whiteflies were placed on each explant and left to proliferate. After 21 days of feeding on the virus-infected *N.b.* (ASKO), whiteflies were propagated onto fresh *N.b.*(ASKO) plants. The presence of the virus in the fresh plant was confirmed via DNA extraction and PCR.

### Viral DNA extraction & PCR

Edwards DNA extraction^56^ and PCR were used to screen for transmission of the A- and B-component of the bipartite tomato mottle virus (ToMoV). A positive control for *N.b.*(ASKO) plant DNA extraction was confirmed based on a 112-bp amplicon of the kanamycin resistance/NptII gene (FWD- TTGCCGAATATCATGGTGGA and REV- TCAGCAATATCACGGGTAGC), which gave a consistent but weak band. The presence of virus was based on amplicons for the ToMoV A-component coat protein (CP), B-component BR1 movement protein (MP) and viral REP gene: a 227-bp amplicon for CP gene (FWD- CGTGCAGGTATTGGGCCAAG and REV- CCAACACGGTGGGTTATGCC), a 719-bp amplicon for the B-component BR1 MP gene (FWD- GTGTTTGACTATGTATCCTTTA and REV- GCCTTGTTAGTTTATTATTGCT), and a 354-bp amplicon from the viral REP gene (FWD- CCCCCACCAAAGAAATTTAGAG and REV- GTCGATTTGGAAATCTCCCC). The viral REP amplicon was gel-purified from a 2% agarose gel and sequenced for confirmation of successful virus transmission.

## Electronic supplementary material

Below is the link to the electronic supplementary material.

Supplementary Material 1

Supplementary Material 2

Supplementary Material 3

Supplementary Material 4

Supplementary Material 5

Supplementary Material 6

Supplementary Material 7

## Data Availability

Some of the raw data (particularly data corresponding to figures and associated statistics) has been deposited in the Penn State Data Commons (www.datacommons.psu.edu) at URL https://doi.org/10.26208/MERD-3Y39. Whiteflies and viral plant pathogen components can be provided only under a USDA-APHIS permit.

## Abbreviations

ASKO: Acyl-sugar knockout
BSL3: Biosafety level 3
BSL2: Biosafety level 2
D: *Dioscorea*
Dpi: Days Post-Infection
GA7: clear, autoclavable 4”x3”x3” tissue culture vessel
MHP: Maintenance Host Plant
MS: Murashige and Skoog media
Nb: *Nicotiana benthamiana*
OD: optical density
PCR: polymerase chain reaction
PDA: potato dextrose agar
PFTE: polytetrafluoroethylene
P-I-V: plant-insect-virus
PTC: plant tissue culture
RIM: Root Induction Media
ToMoV: tomato mottle virus
YBM: yam basic medium
