# Publisher Correction: Enabling biocontained plant virus transmission studies through establishment of an axenic whitefly (*Bemisia tabaci*) colony on plant tissue culture

**DOI:** 10.1038/s41598-024-81406-x

**Published:** 2024-12-03

**Authors:** Natalie S. Thompson, David Krum, Yun-Ru Chen, Mariela C. Torres, Marena A. Trauger, Dalton Strike, Zachary Weston, Jane E. Polston, Wayne R. Curtis

**Affiliations:** 1 Department of Chemical Engineering, The Pennsylvania State University, University Park, PA 16802 USA; 2 Department of Biochemistry and Molecular Biology, The Pennsylvania State University, University Park, PA 16802 USA; 3 Department of Plant Pathology, University of Florida, Gainesville, FL 32611 USA; 4 Intercollege Program in Plant Biology, The Pennsylvania State University, University Park, PA 16802 USA

Correction to: *Scientific Reports* 10.1038/s41598-024-73583-6, published online 15 November 2024

In the original version of this Article a previous rendition of Figure 3 and Figure 4 was published. The original Figure 3 and 4 and accompanying legends appear below.

**Fig. 3:**
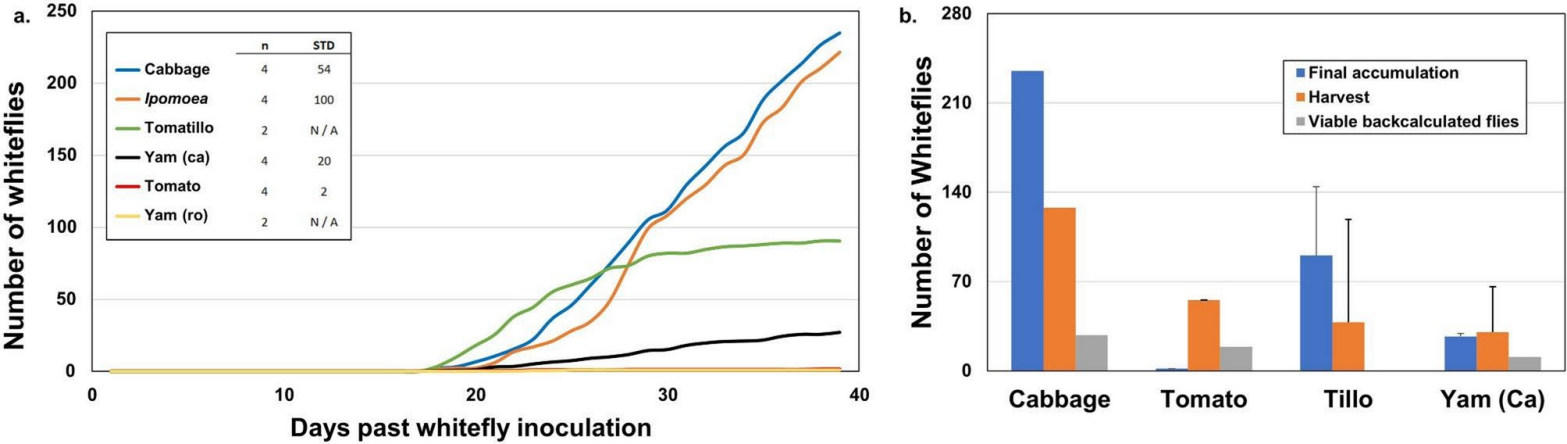
Quantitative assessment of alternative axenic whitefly host plants. (**a**) Daily accumulation based on removal from upper GA7 chamber after inoculation with 20 whiteflies on day zero. Accumulation graphs represent average of replicated experiments as provided in the figure legend. The shaded ‘confidence envelopes’ were generated based on smooth curve fit to the individual time points (α = 0.33 confidence interval corresponds to +/- standard deviation; see DataCommons file). (**b**) Total whiteflies removed during the accumulation study (black bar) and final remaining harvest counts (gray bar, n-1) for cabbage, tomato, and yam (*Dioscorea cayenensis*) and sweet potato (*Ipomoea batatas*). The number of endpoint viable whiteflies at the time of harvest (white bar overlay) is back calculated from the previously published whitefly proliferation model with further detail in Supplemental Material 3 (supplemental Fig. S1.7). Error bars are one standard deviation calculated from n-1 treatments due to one treatment being released into the cabbage viability proliferation and viability back-calculation. *Total whitefly accumulation (accumulation + harvest) is not statistically different on cabbage and sweet potato, but statistically different (**) for tomato (p = 0.0078) and yam (*Dioscorea cayenensis*, p = 0.0079).

**Fig. 4:**
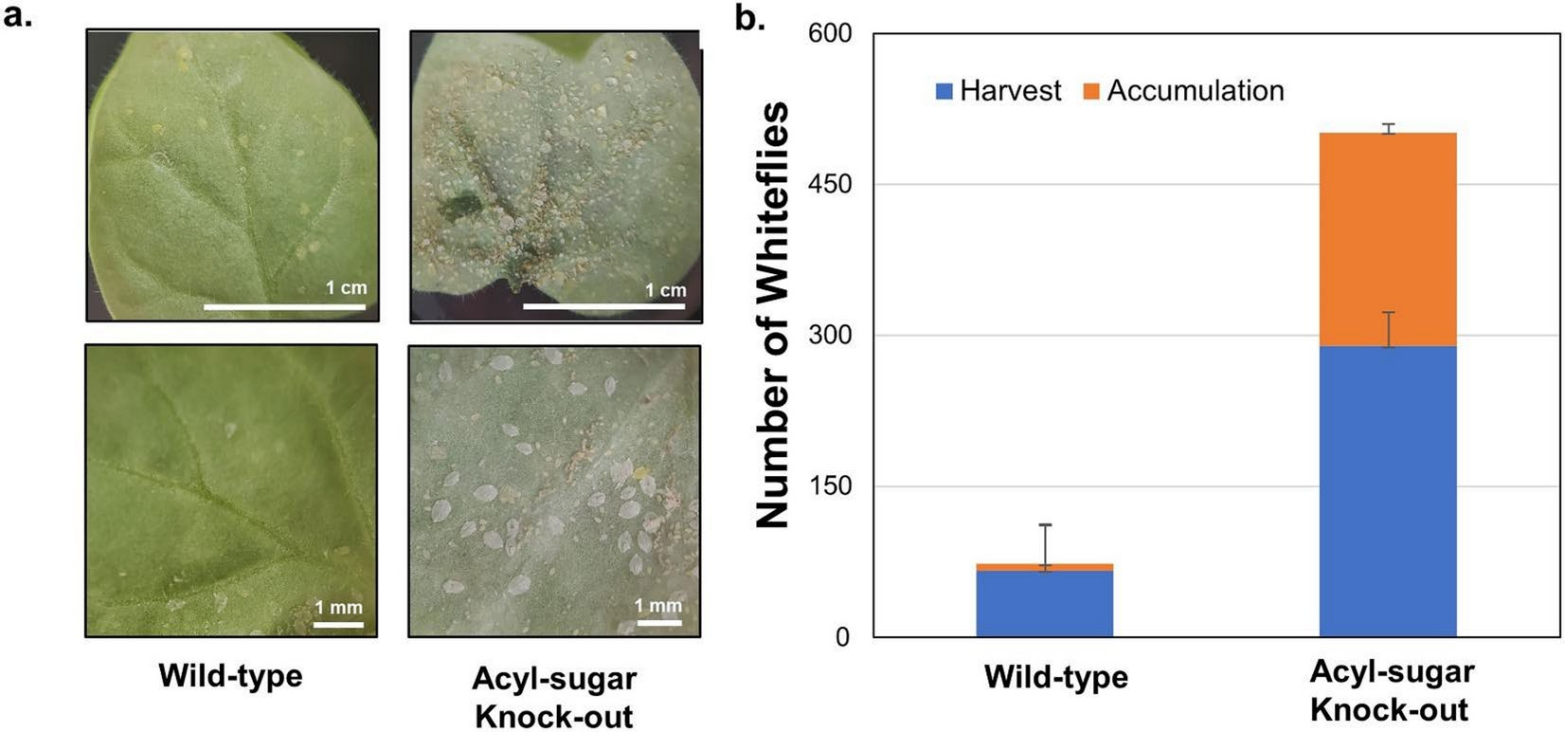
Demonstration of improved whitefly compatibility with model plant *Nicotiana benthamiana* for an acyl-sugar knockout (ASKO). **(a)** Photographic comparison of whitefly proliferation on wild-type and ASKO after 6 weeks of proliferation from 20 initial whiteflies. **(b)** Quantitative comparison of total proliferation measured as the sum of accumulation removed daily from a coupled GA7 and the remainder at harvest endpoint counted by hand. Error bars represent a standard deviation where the improved whitefly proliferation for ASKO was highly statistically significant: whitefly accumulation (*p* = 0.0005), harvest (*p* = 0.0052), and the sum total (*p* = 0.00011).

The original Article has been corrected.

