# Supplementary material for: Enabling biocontained plant virus transmission studies through establishment of an axenic whitefly (Bemisia tabaci) colony on plant tissue culture
Source: Sci Rep. 2024 Nov 15;14:28169. doi: 10.1038/s41598-024-73583-6 (PMC11568280; doi:10.1038/s41598-024-73583-6)
Supplement: Supplementary file 3 — Supplementary Material 3 [file 41598_2024_73583_MOESM3_ESM.pdf]

Enabling biocontained plant virus transmission studies through establishment of an axenic whitefly (Bemisia tabaci) colony on plant tissue culture. Thompson // Curtis

Supplementary Data S1

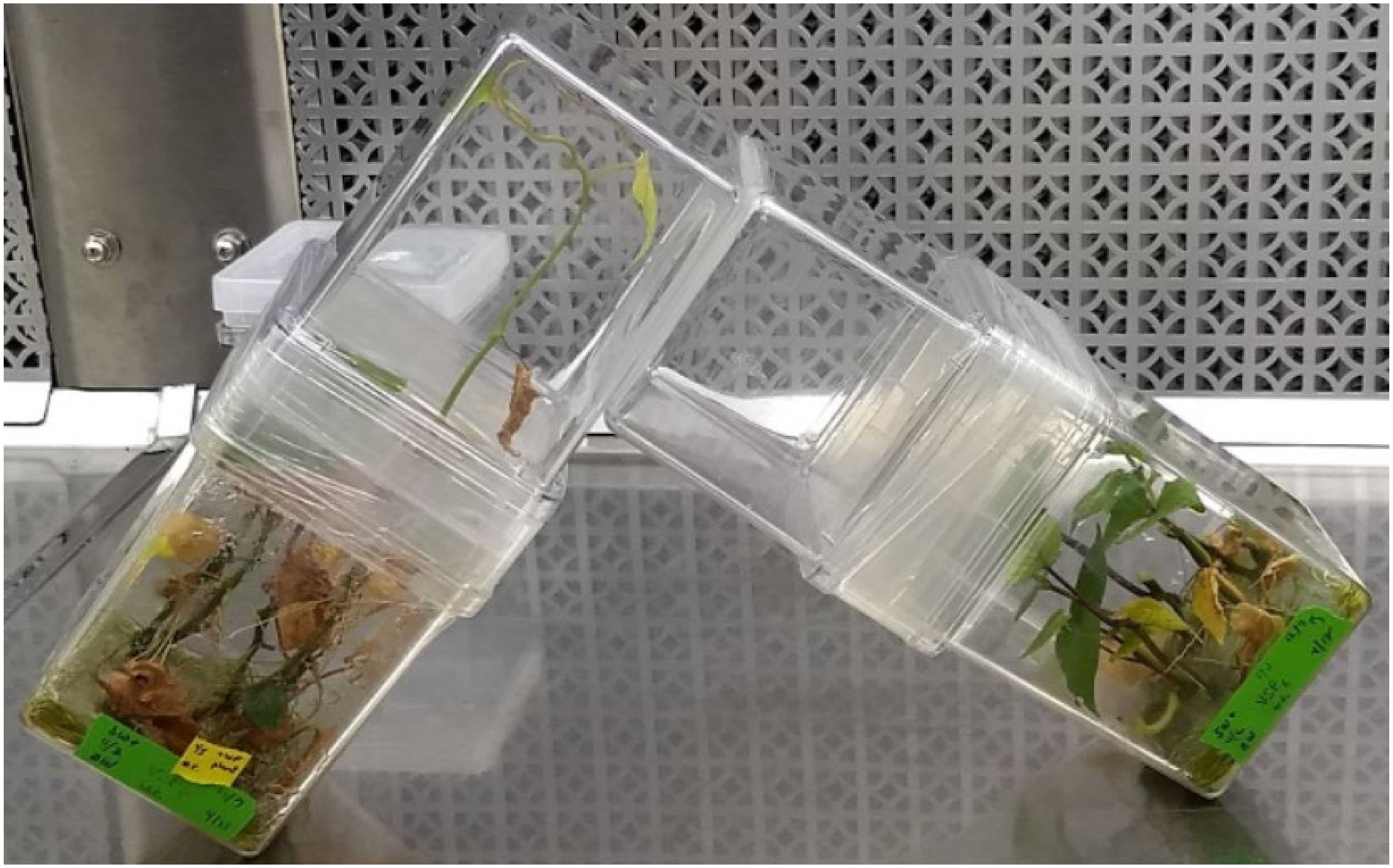

Fig. S1.1: Whitefly plant tissue culture L-Shaped axenic transfer device. Early transfer prototype created by connecting two GA7 couplers together with a hole. The L-shape allows for fresh plants to be swapped in below easily.

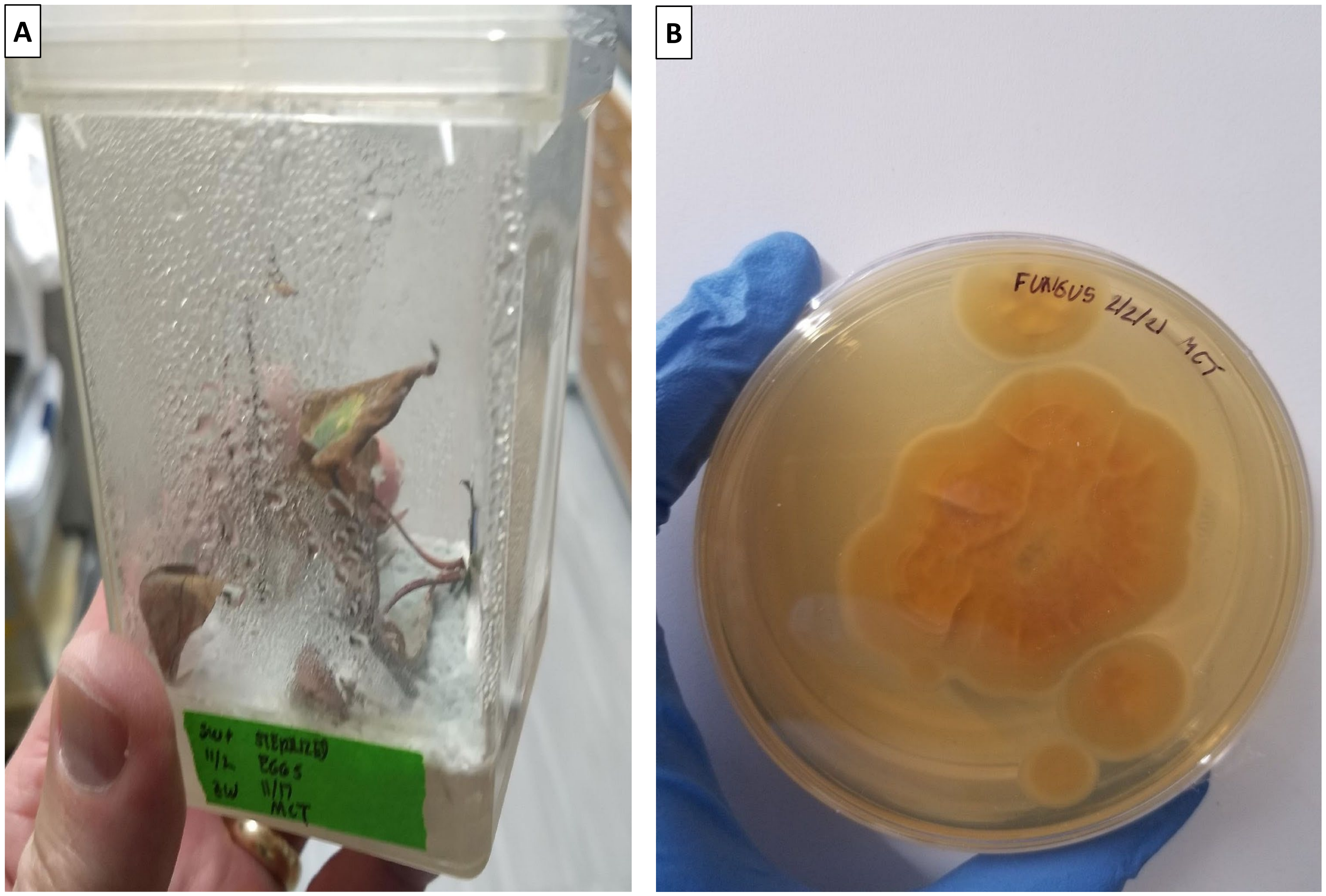

Fig. S1.2: Systemic Fungal Infection. (A) early transfer attempts were prone to fungal infection and as a result later methodology involved ‘outracing’ infection by only letting the whiteflies lay eggs for 1-3 days. (B) Systemic fungus grown on a PDA plate.

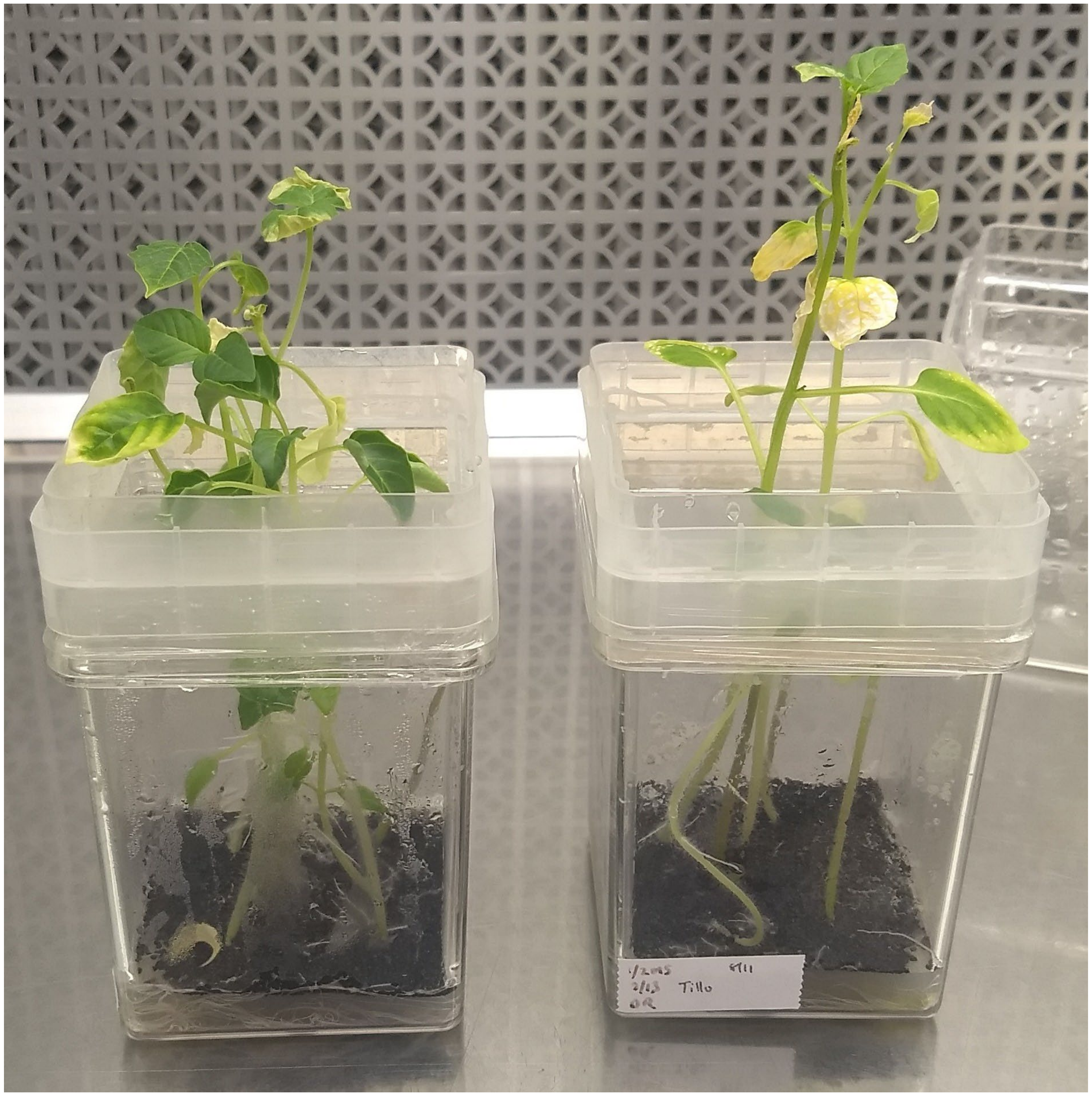

Fig. S1.3: Tomatillo height comparison. Comparison of tomatillo grown on a gyratory shaker (left) and tomatillo grown in normal laboratory conditions (right). Shaking reduced leaf senescence and only mildly reduced the height but the retention of initial leaves resulted in a higher whitefly count.

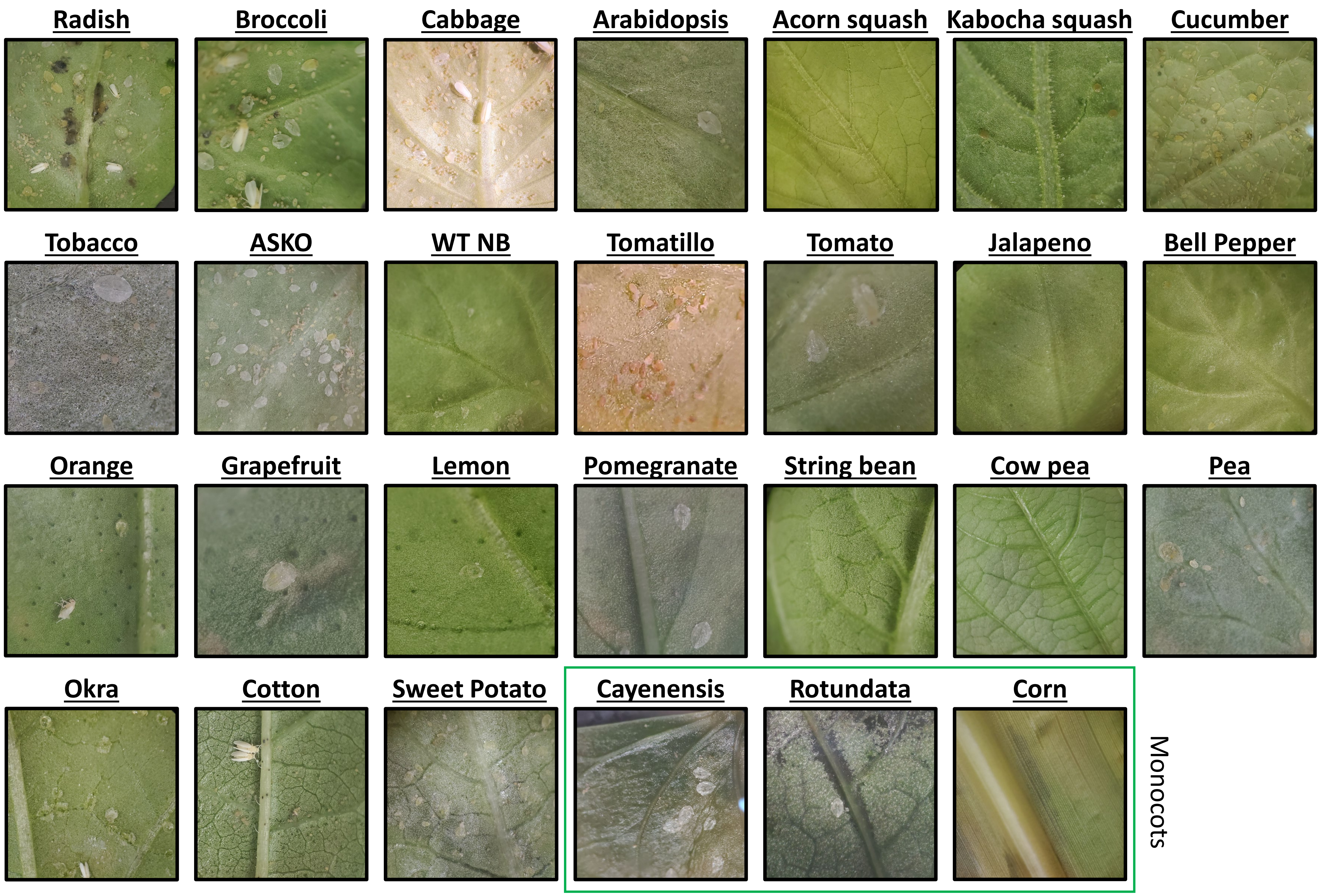

Fig. S1.4: Alternative Host Screen. Close-up shots of the 27 different plant species tested for whitefly proliferation

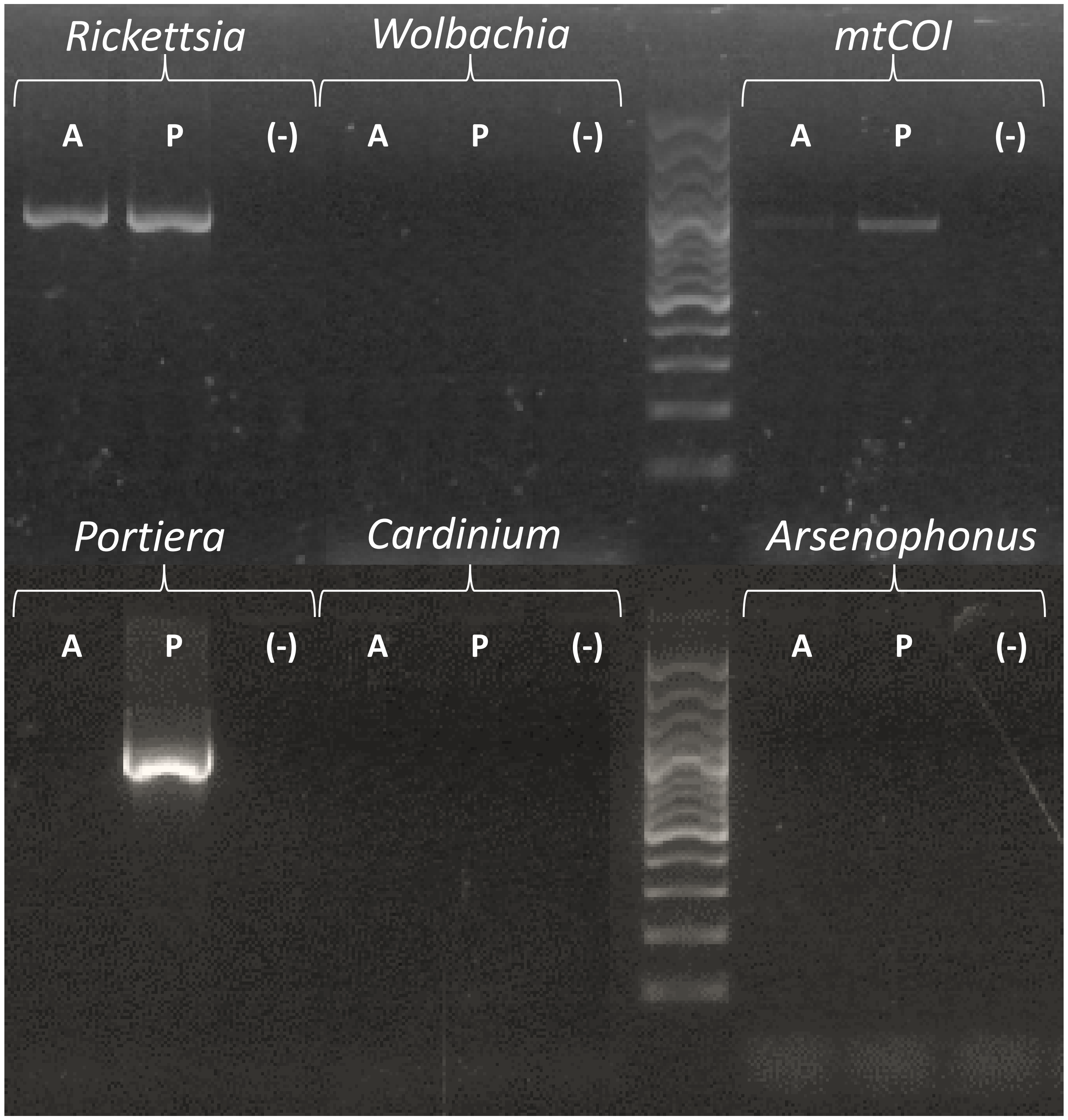

Fig. S1.5. PCR screen for common whitefly endosymbionts in the axenic colony (axenic, A) and our non-axenic (potted, P) colony. Both colonies were negative for *Wolbachia, Cardinium, and Arsenophonus.* The potted colony contains *Portiera* but fell below detection limits in the axenic colony. Both contain *Rickettsia* and were positive for the "control" gene, mtCOI.

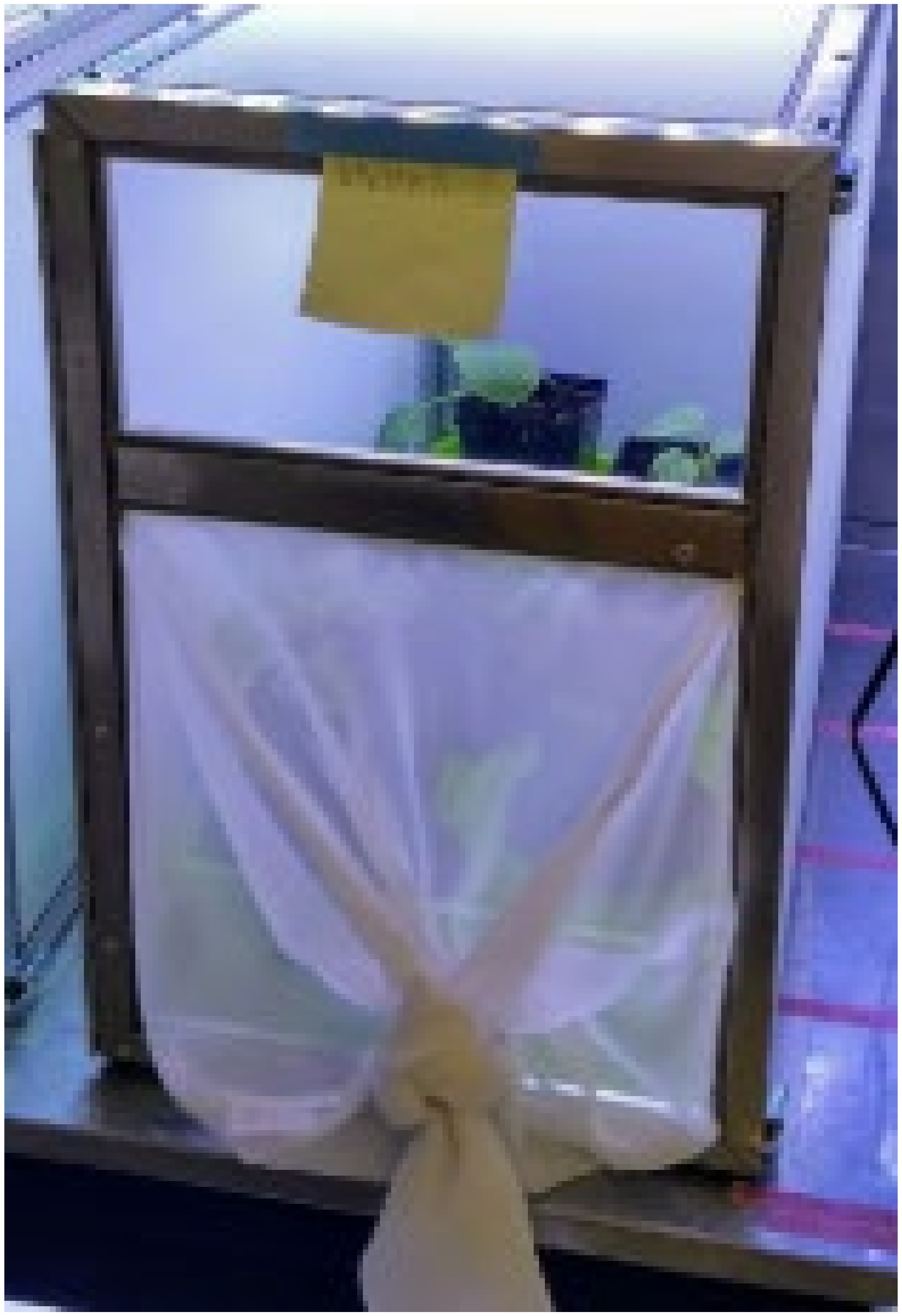

Fig. S1.6: Screen cage with plant flat to provide biocontainment of whiteflies. Cage used to house our potted whitefly colony as well as carry out quantitative proliferation studies as used for back-calculation of viability of a whitefly inoculation.

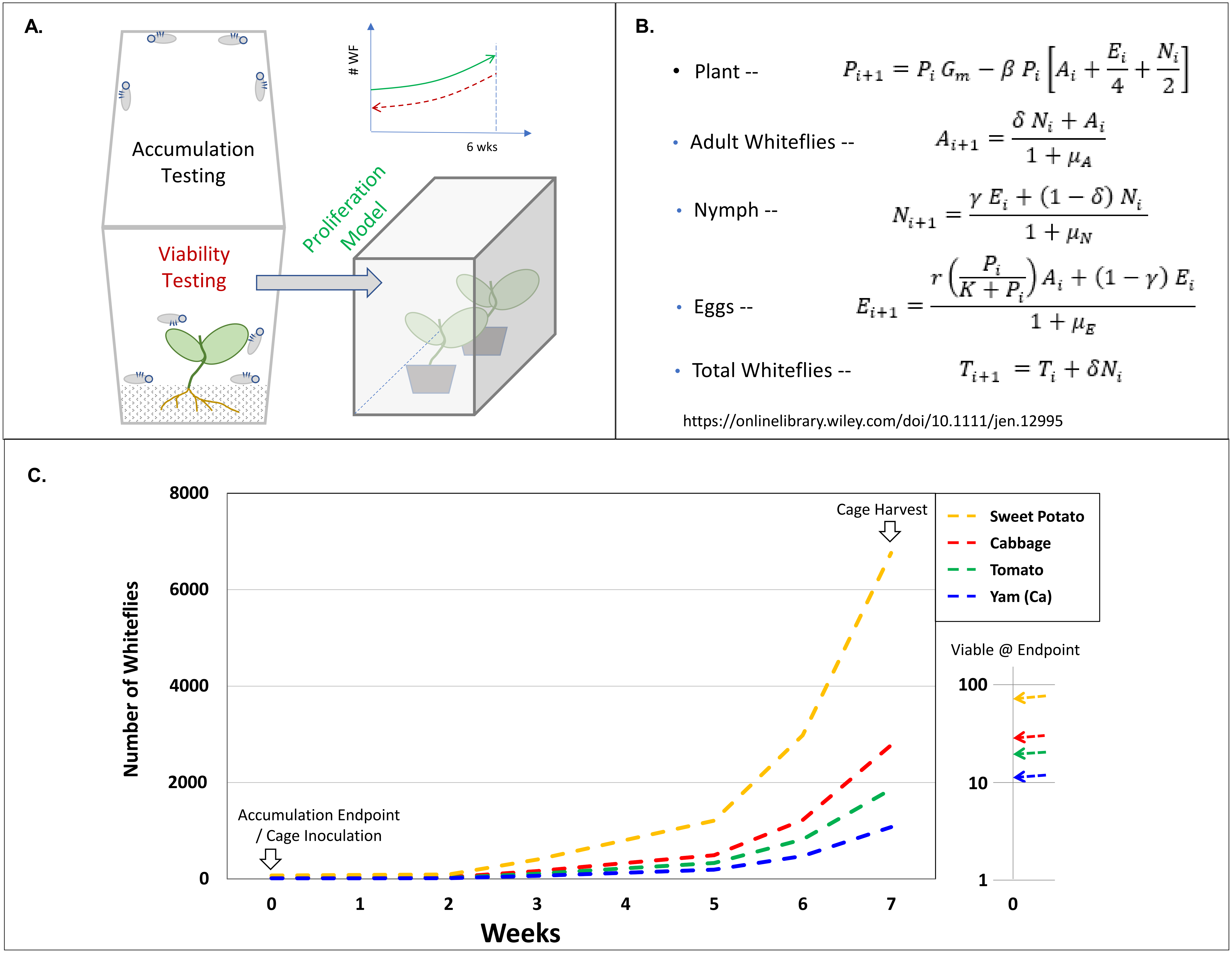

Fig. S1.7: Viability Back-Calculation Using WF Proliferation Model. A. Schematic of releasing a harvest endpoint whitefly GA7 culture into a cabbage proliferation insect cage. **B.** Whitefly proliferation model as developed in citation provided. **C.** Based on endpoint whiteflies harvested, the initial viable flies at the time of inoculation are back-calculated.

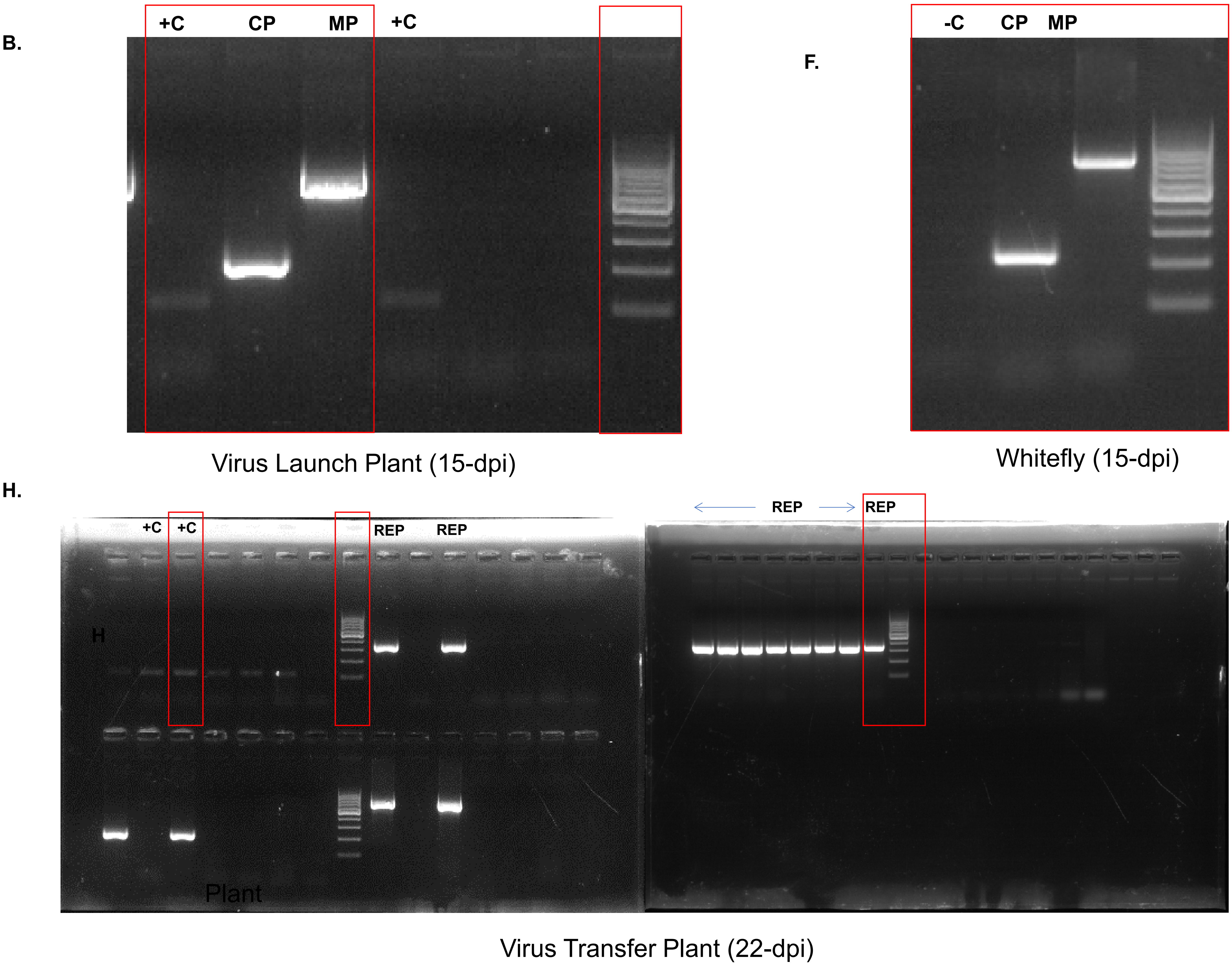

Fig. S1.8: Original gels for Basis of Figure 6 confirming systemic viral infection and viruliferous whitefly.
For Figure 6B. Virus launch plant 15 days after syringe infiltration at a distal leaf to demonstrate systemic infection: CP = coat protein, MP = movement protein, and C+ weakly positive control for transgenic plant selectable marker for kanamycin resistance (NptII).
F. Whitefly were screened by PCR for the presence of the virus; C+ negative control for absence of transgenic plant NptII.
H. 7 days later, leaf samples were PCR screened for the presence of the virus;
