# Supplementary material for: Enabling biocontained plant virus transmission studies through establishment of an axenic whitefly (Bemisia tabaci) colony on plant tissue culture
Source: Sci Rep. 2024 Nov 15;14:28169. doi: 10.1038/s41598-024-73583-6 (PMC11568280; doi:10.1038/s41598-024-73583-6)
Supplement: Supplementary file 4 — Supplementary Material 4 [file 41598_2024_73583_MOESM4_ESM.pdf]

Enabling biocontained plant virus transmission studies through establishment of an axenic whitefly (Bemisia tabaci) colony on plant tissue culture. Thompson // Curtis

Supplementary Data S3

Axenic Whiteflies - Alternative Host - Data Sheet

Plant	Picture (top)	Picture (side)	Plant picture	Observations	Optimal Seed #
Cabbage	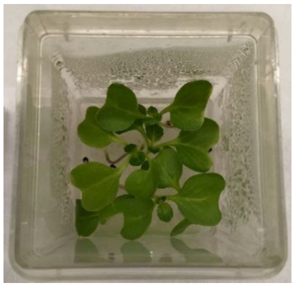	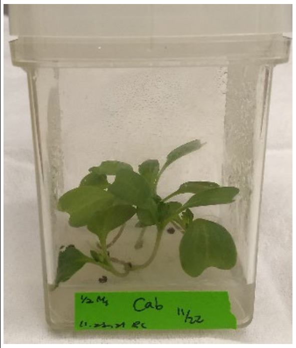	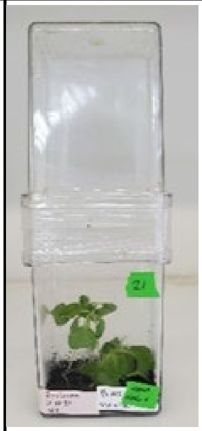	Great size for growth in tissue vessel. Leaf senescence happens very slowly.	4-6
Broccoli	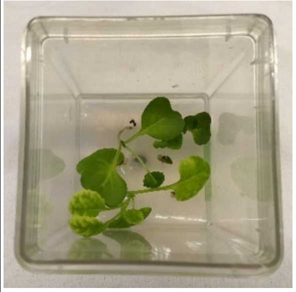	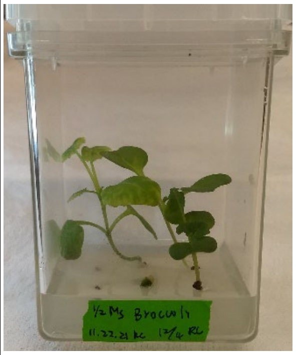	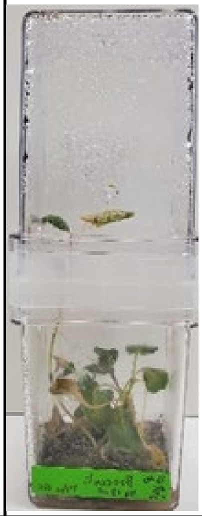	Great size for growth in tissue vessel. Leaf senescence happens very slowly.	4-6
Cotton	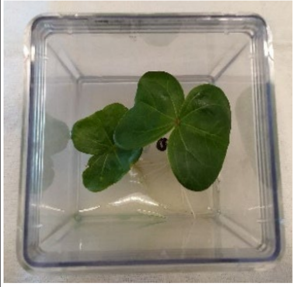	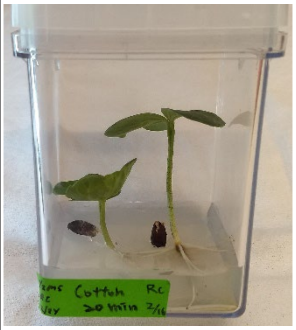	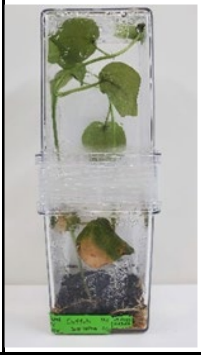	Very large leaves which were initially good for whiteflies but it quickly outgrew the two GA7 combined height	2

Corn	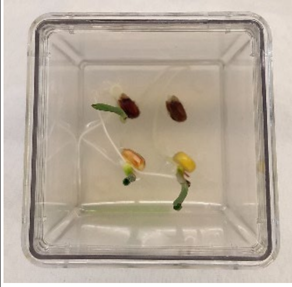	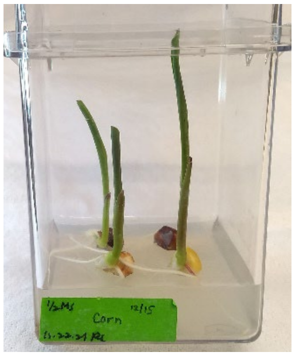	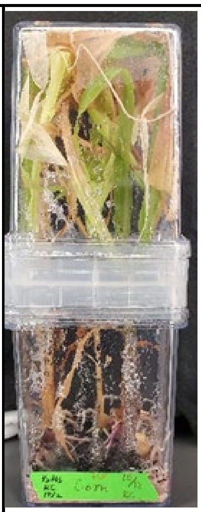	Quickly outgrew the height of the two GA7's. Monocot.	2-4
Cucumber	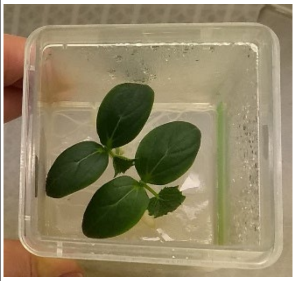	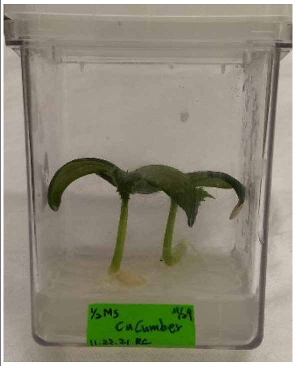	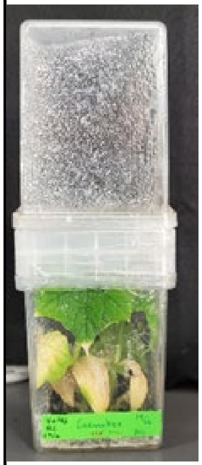	Nice compact, large cotyledon growth	2
Leopard Melon	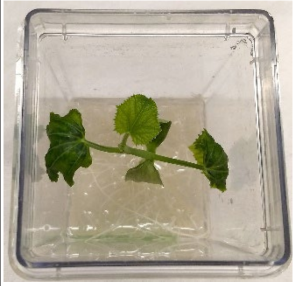	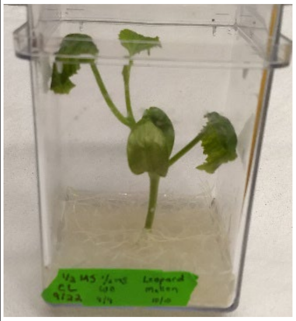		Compact, nice leaf size.	1

Acorn Squash	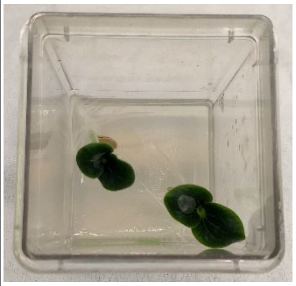	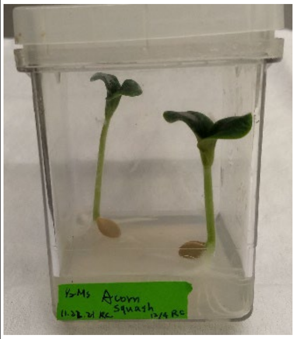	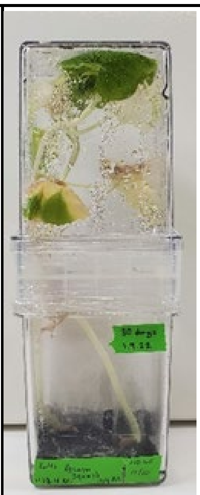	Vine, outgrew GA7's quickly	2
Kabocha	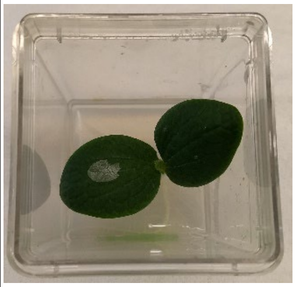	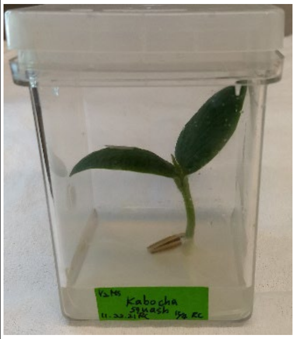	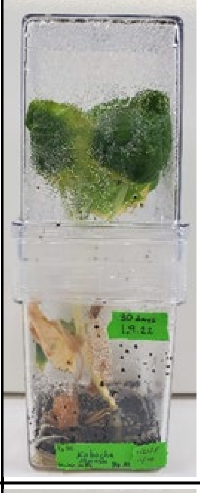	Great cotyledon size and leaf area.	1
Pea	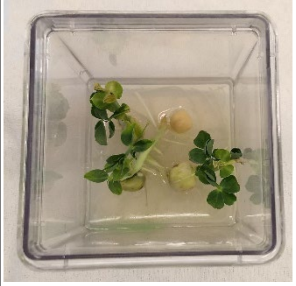	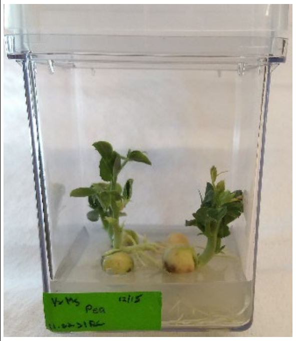	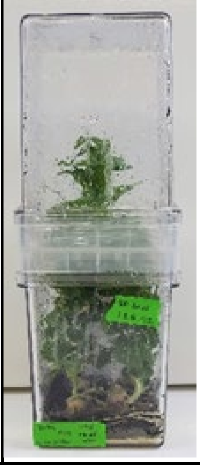	Small leaves, worked well as a whitefly host.	3-4

CowPea	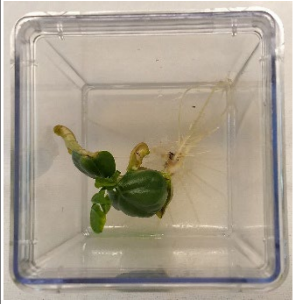	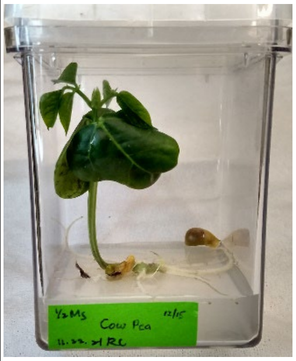	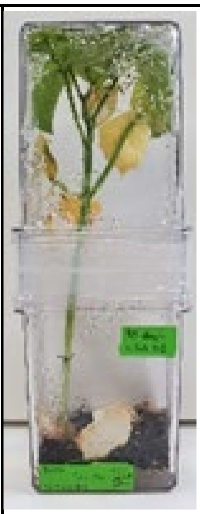	Grew far to quickly and too tall but the leaves were a great size.	1
Soy	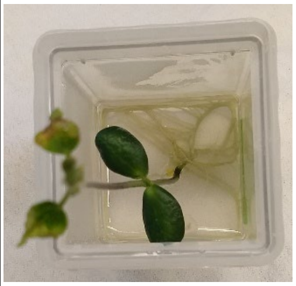	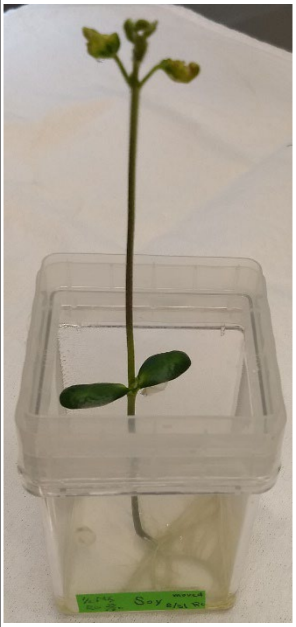		Grew very high very quickly. Small leaves	1
String Bean	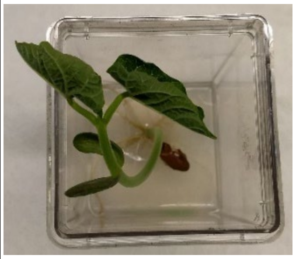	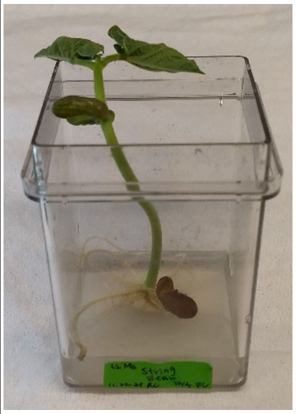	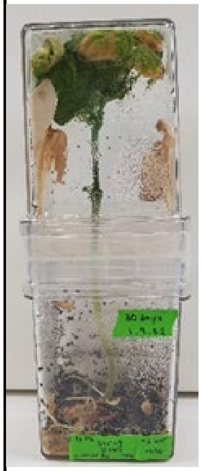	Grew far to quickly and too tall but the leaves were a great size.	1

Bell Pepper	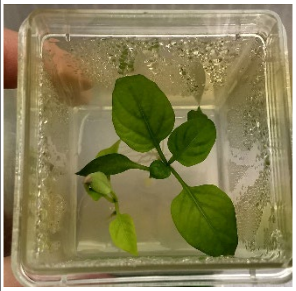		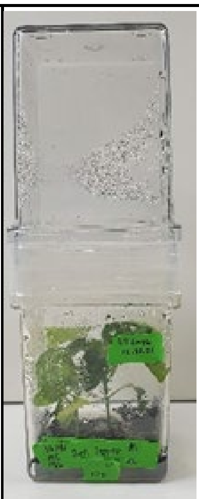	Slow grower. Great final plant size	2-3
Jalapeno	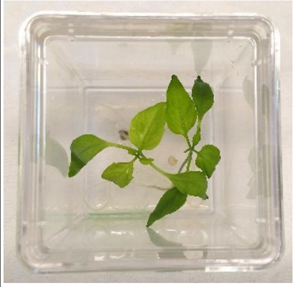	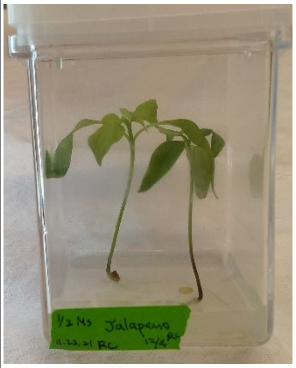	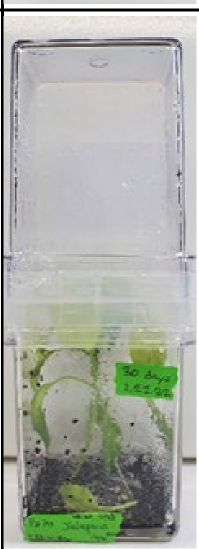	Narrow leaves but good final plant size.	2-4
Tomato	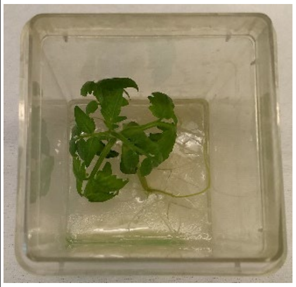	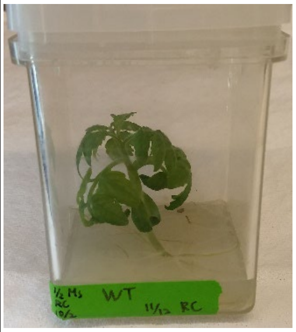	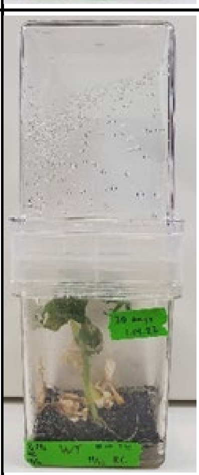	One plant per GA7 minimized the leaves curling and allowed larger expansion. Trichomes were hard for the whiteflies to feed through.	1

Nicotiana tabacum	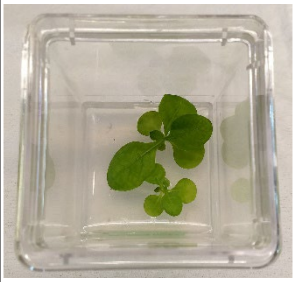	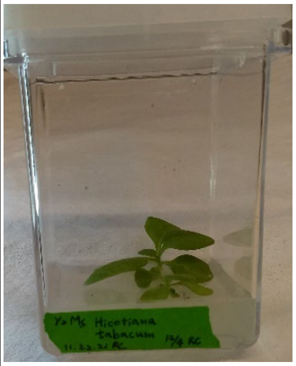	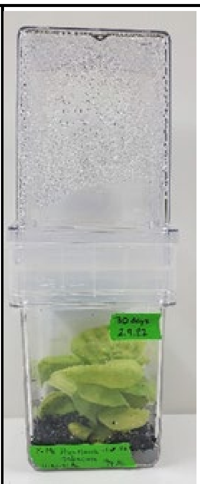	Great leaf and final plant size	2-5
Nicotiana benthamiana	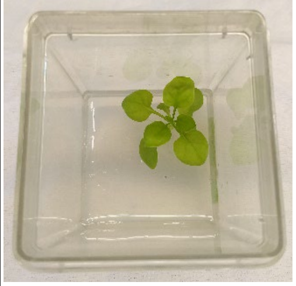	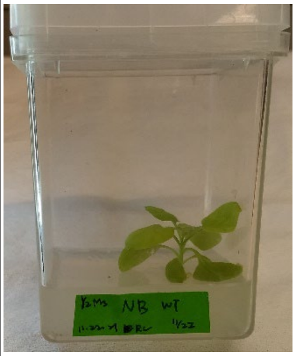	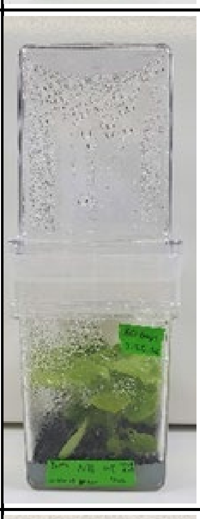	Great leaf and final plant size	2-5
Acyl-sugar knockout *Nicotiana benthamiana*	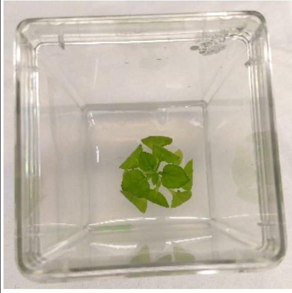	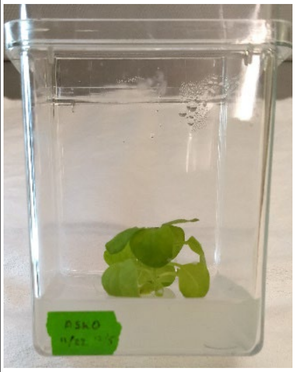	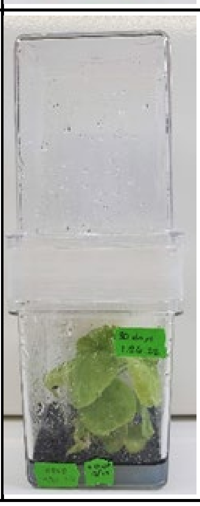	Great leaf and final plant size. The acyl-sugar knockout variety had much larger fecundity of the whiteflies.	2-5

Okra	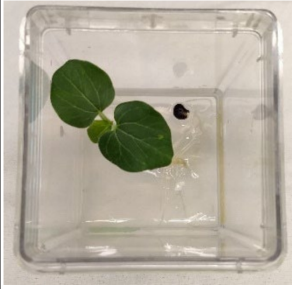	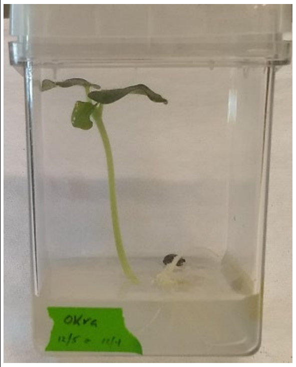	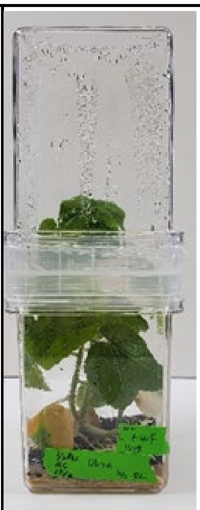	Roots pushed the seed out of the media but the final plant size was good.	1
Radish	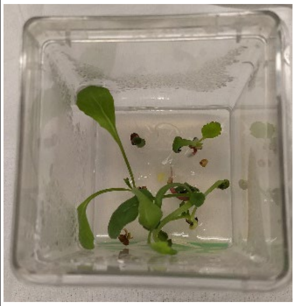	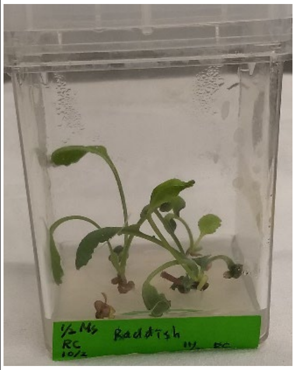	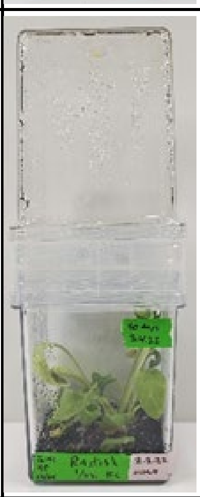	Narrow leaves but height of the plant was good.	2-5
Sweet Potato	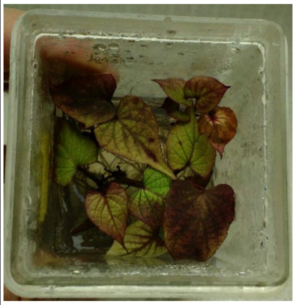	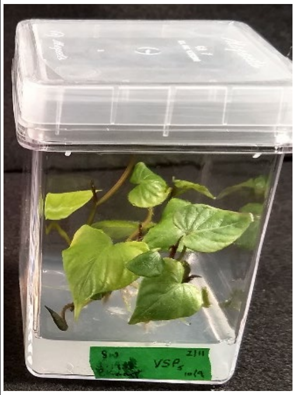		Great in tissue culture. Needs to be subcultured once every couple of months.	3-4

Yam (Ro)	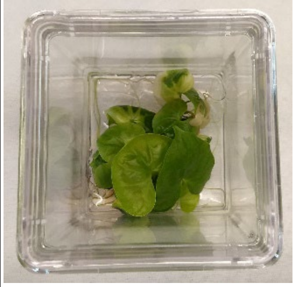	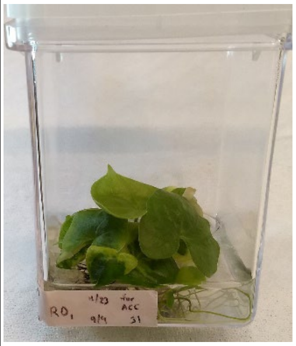	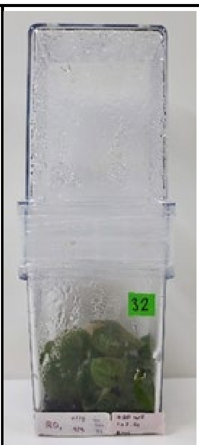	Monocot (Dioscorea); Great in tissue culture. Needs to be subcultured once every couple of months.	3-4
Yam (Ca)	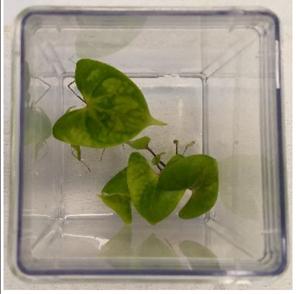	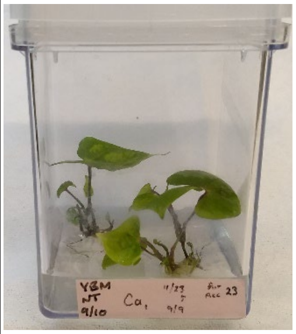	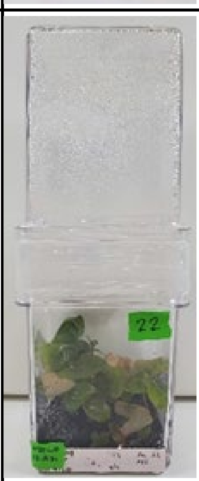	Monocot (Dioscorea); Great in tissue culture. Needs to be subcultured once every couple of months.	3-4
Greenbriar	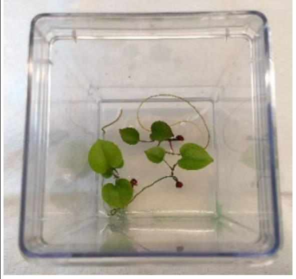	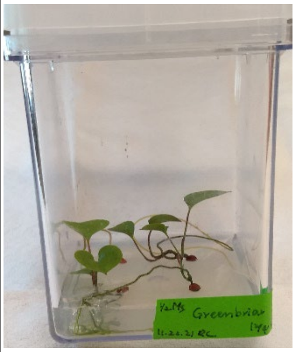		Struggled with germination. It grew very slowly and leaf size was very small; Monocot	4

Orange	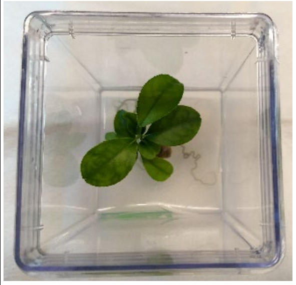	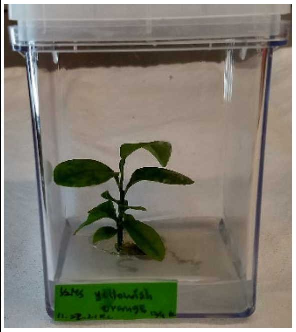	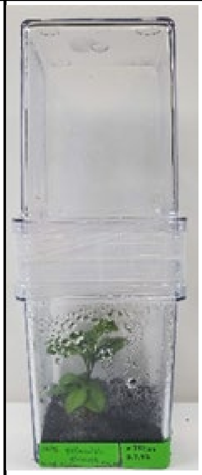	Great leaf size and height.	1-2
Lemon	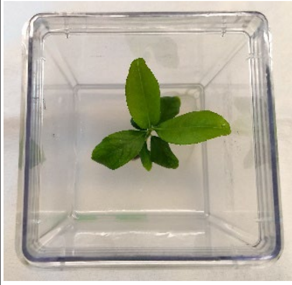	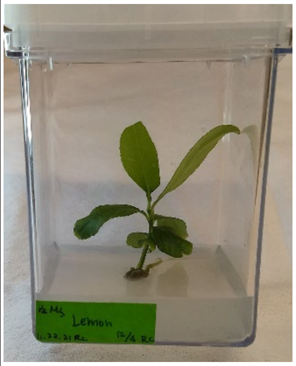	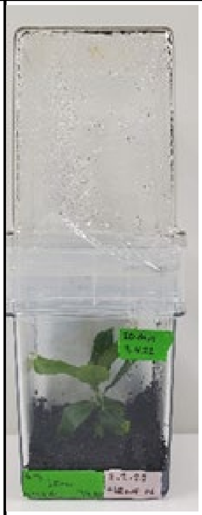	Great leaf size and height.	1-2
Grapefruit	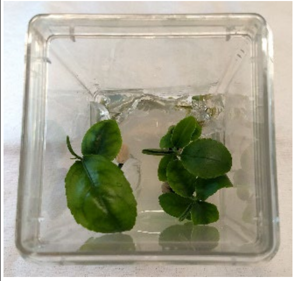	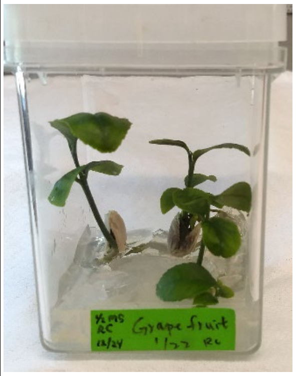	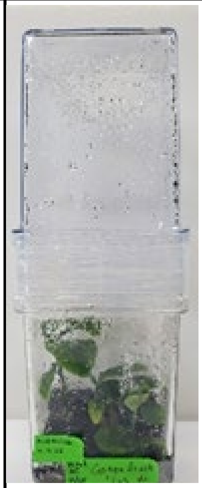	Great leaf size and height.	1-2

Arabidopsis	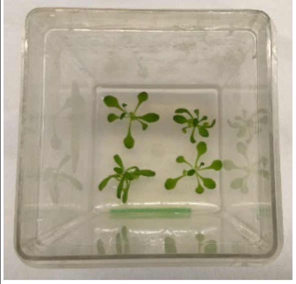	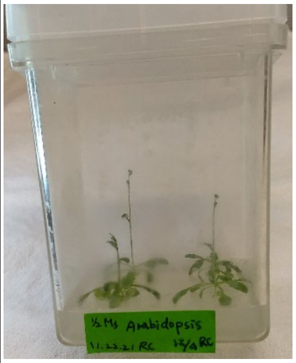	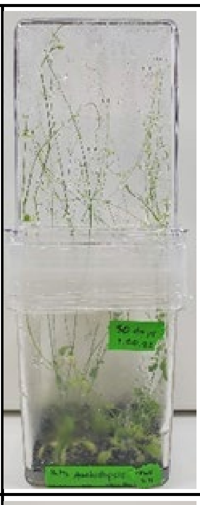	Very small leaves did not work for whiteflies in tissue culture and it bolted quickly which was too high for the GA7 combo.	4
Pomegranate	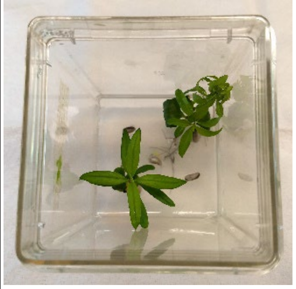	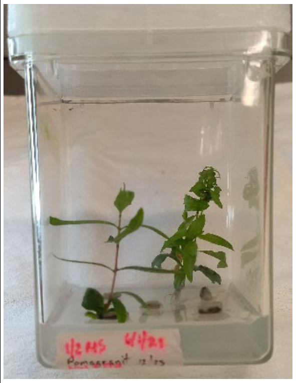	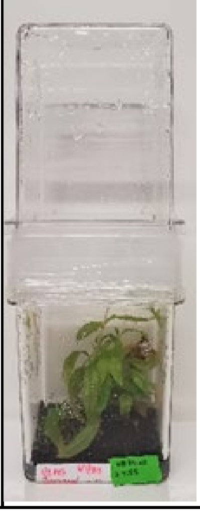	Great height but leaves were very narrow.	1-2
