# Supplementary material for: Enabling biocontained plant virus transmission studies through establishment of an axenic whitefly (Bemisia tabaci) colony on plant tissue culture
Source: Sci Rep. 2024 Nov 15;14:28169. doi: 10.1038/s41598-024-73583-6 (PMC11568280; doi:10.1038/s41598-024-73583-6)
Supplement: Supplementary file 5 — Supplementary Material 5 [file 41598_2024_73583_MOESM5_ESM.pdf]

Enabling biocontained plant virus transmission studies through establishment of an axenic whitefly (*Bemisia tabaci*) colony on plant tissue culture. Thompson // Curtis

Supplementary Data S4

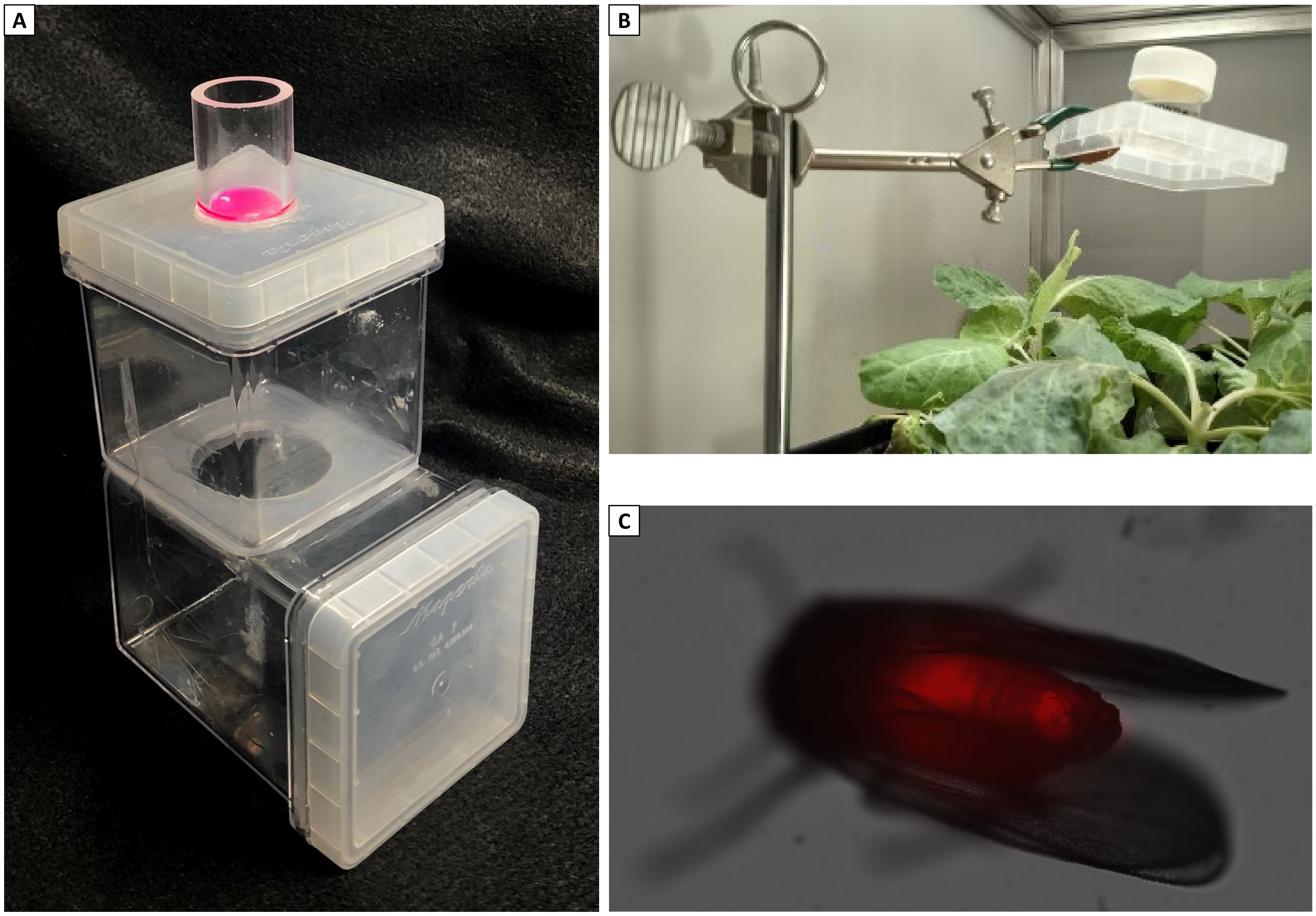

Fig. S4.1: Membrane Feeding Assemblies for Feeding Whitefly and Confocal Fly Image. A. Feeding assembly build with PTFE membrane cut-out under 1” acrylic tube inserted onto lid of GA7. Whiteflies were added to the central containers and dyed sucrose solution was added on-top of the membrane. **B.** Prototype testing of feeding with non-axenic whiteflies. **C.** Microscopic whitefly image of after fluorescent dye feeding (bright-field overlay of fluorescent imaging on a DIY Thor fluorescent microscope platform).

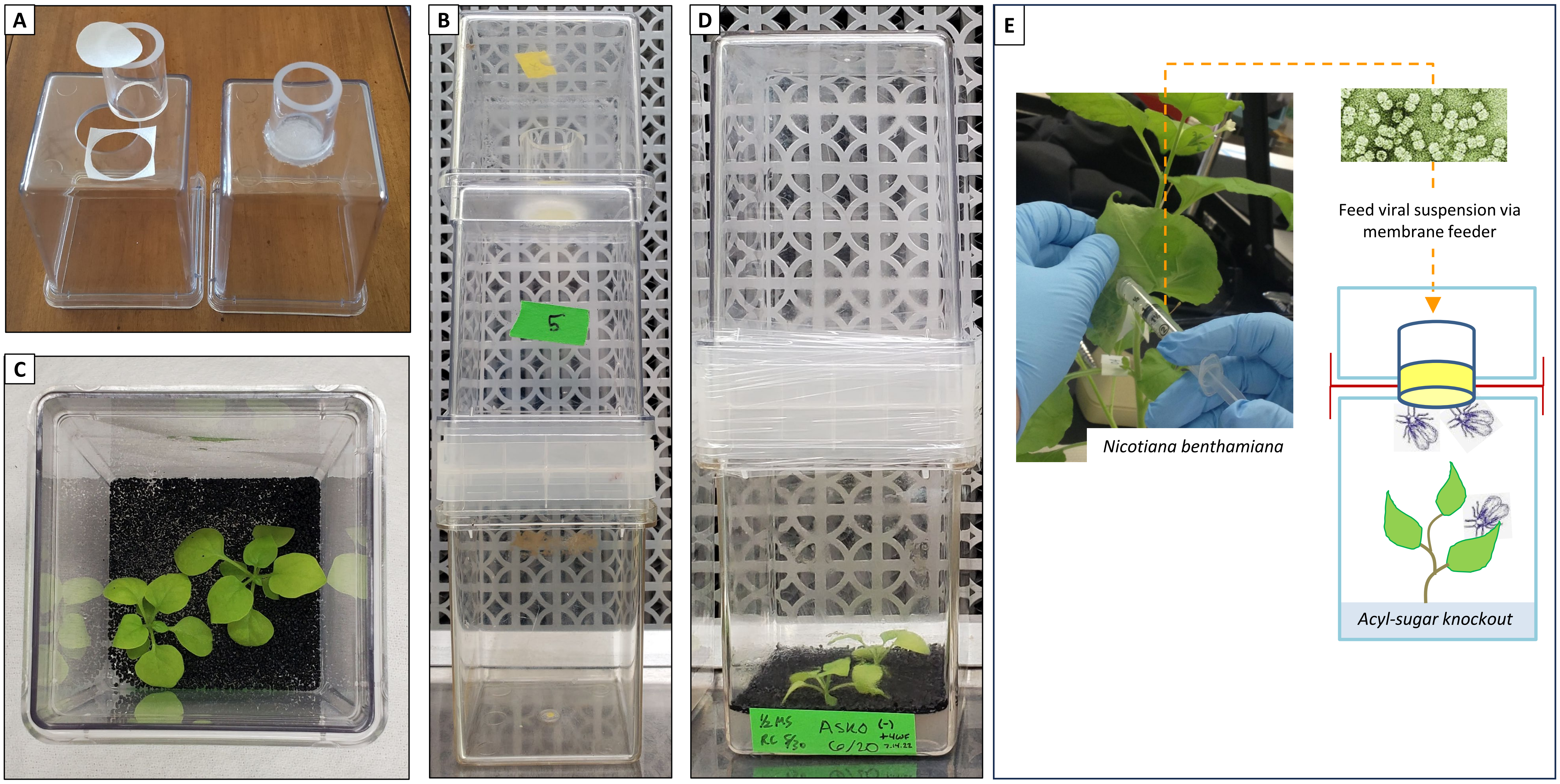

Fig. S4.2: Virus suspension feeding and transmission overview. A. Feeding assembly build with PTFE membrane cut-out under 1” acrylic tube inserted through polycarbonate GA7. **B.** Whiteflies feeding on filer-sterilized virus suspension in buffer and sucrose solution (and yellow food coloring to attract WF). top inverted GA7 slows evaporation of virus suspension. **C.** Picture of host *N. benthamiana* (ASKO) plants after condensation is removed and autoclaved black sand added to prepare for addition of whiteflies. **D.** ASKO host plant with virus containing whiteflies added for virus transmission. **E.** Overall approach including launch of the virus(es) by Agro-infiltration, followed by prep of virus and then transmission to tissue cultured plants through membrane feeding.
