# Supplementary material for: Enabling biocontained plant virus transmission studies through establishment of an axenic whitefly (Bemisia tabaci) colony on plant tissue culture
Source: Sci Rep. 2024 Nov 15;14:28169. doi: 10.1038/s41598-024-73583-6 (PMC11568280; doi:10.1038/s41598-024-73583-6)
Supplement: Supplementary file 6 — Supplementary Material 6 [file 41598_2024_73583_MOESM6_ESM.pdf]

Supplementary Data 6

SWeet Potato Shoot Medium (**SW**)

	[final]	[stock]	prep / L	/ 250 mL
**Sucrose**	30 g/L	--	30 g	7.5 g
**MS Macronutrients (10x)**			100 mL	25 mL
NH_4NO_3 (ammonium nitrate)	1.65 g/L	16.5 g/L		
KNO_3 (potassium nitrate)	1.9 g/L	19 g/L		
$CaCl_2{\cdot}2H_20$ (A)	0.44 g.L	4.4 g/L (A)		
$MgSO_4{\cdot}7H_20$ (B)	0.37 g/L	3.7 g/L (B)		
KH_2PO_4 (potassium phosphate monobasic)	0.17 g/L	1.7 g/L		
**MS Micronutrients (1000x)**			1 mL	0.25 mL
$MnSO_4{\cdot}H_2O$ (C) (manganous sulfate)		16.9 g/L (C)		
$ZnSO_4{\cdot}7H_20$		8.6 g/L		
H_3BO_3 (boric acid)		6.2 g/L		
KI (potassium iodide)		0.83 g/L		
$Na_2MoO_4{\cdot}2H_20$ (sodium molybdate)		0.25 g/L		
$CuSO_4{\cdot}5H_20$ (D)		0.025 g/L (D)		
$CoCl_2{\cdot}6H_20$		0.025 g/L		
**Fe-EDTA**·[$2H_20$] (E) (iron-EDTA)	40.3 mg / L	4.0 g/L (4.0 mg/mL)	10 mL	2.5 mL
**MS vitamins** Thiamine-HCl	0.1 mg/L	0.1 g/L (0.1 mg/mL)	1 mL	0.25 mL
Nicotinic acid	0.5 mg/L	0.5 g/L (0.5 mg/mL)		
Pyridoxine	0.5 mg/L	0.5 g/L (0.5 mg/mL)		
**Inositol**	100 mg/L	10 g/L (10 mg/mL)	10 mL	2.5 mL
**Glycine**	2 mg/L	0.2 g/L (0.2 mg/mL)	10 mL	2.5 mL
**Kinetin**	0.5 mg/L	50 mg/L (.05 mg/mL)	10 mL	2.5 mL
pH = 5.7 (record initial pH)				
**Agar (granulated 'cheep' stuff)**	6 g/L		6 g	1.5 g
Add BAP from frozen stocks after Autoclaving 30 min				
**BAP** (6-benzylaminopurine)	0.5 mg/L	10 g/L (10 mg/mL)	50 µL	12.5 µL

Typical volume: 40-50 mL / GA7

(A) (dihydrate MW=147) If use anhydrous $CaCl_2$ (MW=111), stock contains 3.32 g/L

(B) (heptahydrate MW=246.5) If use anhydrous $MgSO_4$ (MW=120), stock contains 1.81 g/L

(C) (manganese II sulfate, monohydrate MW=169.0) If use anhydrous $MnSO_4$ (MW=151), stock contains 15.1 g/L. Many older formulations specify $MnSO_4{\cdot}4H_2O$ (could not find in catalogs), stock 22.3 g/L.

(D) (pentahydrate MW=249.7) If use anhydrous $CuSO_4$ (MW=159.6), stock contains 0.016 g/L.

(E) (Ferrous EDTA: $FeNaC_{10}H_{12}N_2O_8$; anhydrous MW=367.1) The original Iron-chelator solution was created by adding 27.3 mg/L $FeSO_4{\cdot}7H_2O$ (FW=278) plus 37.3 mg/L Na_2-EDTA [although the degree of hydration of di-sodium EDTA is not specified on several venders (Sigma, Gibco) the dihydrate (FW=372.2) is the only available form, and this would give the same molarity (0.100 mM) for both Fe and EDTA. The iron-sodium-EDTA is now available, but apparently it has a variable degree of hydration (3 batches - all were different). Equivalent Fe and EDTA molarity can be calculated: Fe-EDTA· [$2H_2O$] (FW=403.1), 4.03 g/L; [·$2.5H_20$] (FW=412.1), 4.12 g/L; [·$3H_20$] (FW=421.1), 4.21 g/L …

Enabling biocontained plant virus transmission studies through establishment of an axenic whitefly (*Bemisia tabaci*) colony on plant tissue culture. Thompson // Curtis

Supplementary Data 6

MS Tomato Root Induction (**MS-TRI**) Medium *for Tomato (FL lanai)*

	[final]	[stock]	prep / L	/ 250 mL
**Sucrose**	30 g/L	--	30 g	7.5 g
**MS Macronutrients (10x)**			100 mL	25 mL
NH_4NO_3 (ammonium nitrate)	1.65 g/L	16.5 g/L		
KNO_3 (potassium nitrate)	1.9 g/L	19 g/L		
$CaCl_2 \cdot 2H_20$ (A)	0.44 g.L	4.4 g/L (A)		
$MgSO_4 \cdot 7H_20$ (B)	0.37 g/L	3.7 g/L (B)		
KH_2PO_4 (potassium phosphate monobasic)	0.17 g/L	1.7 g/L		
**MS Micronutrients (1000x)**			1 mL	250 µL
$MnSO_4 \cdot H_2O$ (C) (manganous sulfate)		16.9 g/L (C)		
$ZnSO_4 \cdot 7H_20$		8.6 g/L		
H_3BO_3 (boric acid)		6.2 g/L		
KI (potassium iodide)		0.83 g/L		
$Na_2MoO_4 \cdot 2H_20$ (sodium molybdate)		0.25 g/L		
$CuSO_4 \cdot 5H_20$ (D)		0.025 g/L (D)		
$CoCl_2 \cdot 6H_20$		0.025 g/L		
**Fe-EDTA**·[2H₂0] (E) (iron-EDTA)	40.3 mg/L	4.0 g/L (4.0 mg/mL)	10 mL	2.5 mL
**MS vitamins** Thiamine-HCl	0.1 mg/L	0.1 g/L (0.1 mg/mL)	1 mL	250 µL
Nicotinic acid	0.5 mg/L	0.5 g/L (0.5 mg/mL)		
Pyridoxine	0.5 mg/L	0.5 g/L (0.5 mg/mL)		
**Inositol**	100 mg/L	10 g/L (10 mg/mL)	10 mL	2.5 mL
**Glycine**	2 mg/L	0.2 g/L (0.2 mg/mL)	10 mL	2.5 mL
pH = 5.5 (record initial pH)				
Agar for Plant Tissue Culture (0.6%)	0.6 g/L	--	6 g	1.5 g
Autoclave				
**Indole-3-Butyric Acid (IBA)**	0.05 mg/L	25 g/L (25 mg/mL)	2 µL	0.5 µL
**Cefotaxime**	500 mg/L	500 g/L (500 mg/mL)	1 mL	250 µL
Kanamycin (if using NPT selectable marker)	100 mg/L	100 g/L (100 mg/mL)	1 mL	250 µL
Hygromycin (if using HPT selectable marker)	20 mg/L(F)	50 g/L (50 mg/mL)	400 µL	100 µL
