# Supplementary material for: Enabling biocontained plant virus transmission studies through establishment of an axenic whitefly (Bemisia tabaci) colony on plant tissue culture
Source: Sci Rep. 2024 Nov 15;14:28169. doi: 10.1038/s41598-024-73583-6 (PMC11568280; doi:10.1038/s41598-024-73583-6)
Supplement: Supplementary file 7 — Supplementary Material 7 [file 41598_2024_73583_MOESM7_ESM.pdf]

Supplementary Data S7

Primers used to assess presence of whitefly endosymbionts:

Gene	Primer sequence (5’ → 3’)	Amplicon size / T_A	Reference
B. tabaci mtCOI	F: TTGATTTTTTGGTCATCCAGAAGT<br>R: TCCAATGCACTAATCTGCCATATTA	48 °C / 800 bp	Frohlich et al. 1999
P. aleyrodidarum 16S rDNA	F: TGCAAGTCGAGCGGCATCAT<br>R: AAAGTTCCCGCCTTATGCGT	54 °C / 1000 bp	Zchori-Fein and Brown 2002
Cardinium 16S rDNA	F: TACTGTAAGAAATAAGCACCGGC<br>R: GTGGATCACTTAACGCTTTCG	50 °C / 400 bp	Zchori-Fein et al. 2004
Arsenophonus 23S rDNA	F: CGTTTGATGAATTCATAGTCAAA<br>R: GGTCCTCCAGTTAGTGTTACCCAAC	44 °C / 900 bp	Thao and Baumann 2004
Wolbachia 16S rDNA	F: CGGGGGAAAAATTTATTGCT<br>R: AGCTGTAATACAGAAAGTAAA	41 °C / 589 bp	Heddi et al. 1999
Rickettsia sp. 16S rRNA	F: GCTCAGAACGAACGCTATC<br>R: GAAGGAAAGCATCTCTGC	47 °C / 589 bp	Gottlieb et al. 2006
Hamiltonella 16S rDNA	F: TGAGTAAAGTCTGGAATCTGG<br>R: AGTTCAAGACCGCAACCTC	47 °C / ~700 bp	Pan et al. 2012
Fritschea 23S rDNA	F: GATGCCTTGGCATTGATAGGCGATGAAGGA<br>R: TGGCTCATCATGCAAAAGGCA	54 °C / ~600 bp	Pan et al. 2012
